# Association between triglyceride glucose index and risk of cerebrovascular disease: systematic review and meta-analysis

**DOI:** 10.1186/s12933-022-01664-9

**Published:** 2022-11-02

**Authors:** Feifei Yan, Shoumeng Yan, Jing Wang, Yani Cui, Feinan Chen, Fang Fang, Weiwei Cui

**Affiliations:** 1 Department of Nutrition and Food Hygiene, School of Public Health, Jilin University, 1163 Xinmin Avenue, 130021 Changchun, P. R. China; 2 School of Nursing, Jilin University, 130021 Changchun, P. R. China

**Keywords:** Triglyceride glucose index, Cerebrovascular disease, Observational research, meta‒analysis, Dose‒response relationship

## Abstract

**Background:**

The triglyceride glucose (TyG) index, which is a new surrogate indicator of insulin resistance (IR), is thought to be associated with many diseases, such as cardiovascular disease, but its relationship with cerebrovascular disease is still controversial.

**Methods:**

The PubMed, EMBASE, Cochrane Library, Web of Science and Medline databases were searched until March 2022 to evaluate the association between the TyG index and cerebrovascular disease risk. A random‒effects model was used to calculate the effect estimates and 95% confidence intervals (CIs).

**Results:**

A total of 19 cohort studies and 10 case‒control/cross‒sectional studies were included in our study, which included 11,944,688 participants. Compared with a low TyG index, a higher TyG index increased the risk of cerebrovascular disease (RR/HR = 1.22, 95% CI [1.14, 1.30], *P*<  0.001; OR = 1.15, 95% CI [1.07, 1.23], *P*<  0.001). Furthermore, the results of the dose-response analysis of the cohort study demonstrated that the risk of cerebrovascular disease increased by 1.19 times per 1 mg/dl increment of the TyG index (relative risk = 1.19, 95% CI [1.13,1.25], *P*<  0.001).

**Conclusion:**

TyG index is related to cerebrovascular disease. More data and basic research are needed to confirm the association.

**Supplementary Information:**

The online version contains supplementary material available at 10.1186/s12933-022-01664-9.

## Introduction

Cerebrovascular disease, which is a general term for a class of diseases caused by pathological changes in cerebral blood vessels leading to brain dysfunction (including localized or diffuse cerebral dysfunction caused by various cerebrovascular diseases such as vascular lumen occlusion or stenosis, vascular rupture, vascular malformation, vascular wall damage, or permeability changes), is one of the main causes of death and incapacity worldwide [1]. The medical costs of cerebrovascular disease have been reported to be higher than those of other vascular diseases. Studies have indicated that total health care costs for cerebrovascular disease are expected to triple if preventive measures are not taken in a timely manner [2]. As the principal clinical type of cerebrovascular disease, stroke has elicited an increasing burden to the global health care system [3]. According to the latest report of the Global Burden of Disease Study, stroke accounts for 11.6% of all deaths and remains the second leading global cause of death, as well as being the third most common cause of disability [4]. Despite a 25% decline in global stroke mortality over the past period, the total number of strokes increased by 70.0% year-on-year, the prevalence of stroke increased by 85.0%, mortality increased by 43.0% and there was a 32.0% increase in disability-adjusted life years due to stroke from 1990 to 2019 [4, 5].

Insulin resistance (IR) is a metabolic disorder caused by a damaged tissue responsiveness to insulin stimulation, especially as manifested in the dysfunction of glucose and blood lipid metabolism [6]. Existing studies have confirmed that IR is intimately related to a variety of cerebrovascular diseases or its triggers, including atherosclerosis, carotid artery plaque formation and rupture, carotid intima-media thickening, hyperglycaemia, dyslipidaemia, stroke and coronary artery disease [6–10]. It is well known that the hyperinsulinaemic-euglycaemic clamp test and HOMA-IR can effectively measure IR [11, 12]. However, due to their complicated operations and high costs, the triglyceride-glucose (TyG) index has been proposed as a simple, economical, intuitive and stable surrogate indicator for IR [11, 13]. Some studies have indicated that a high correlation between TyG and the hyperinsulinaemic-euglycaemic clamp test or HOMA-IR exists [14, 15]. Specifically, the TyG index has been shown to be better than HOMA-IR in predicting certain diseases [16].

The TyG index has been reported to be associated with stroke, carotid atherosclerosis, microvascular and macrovascular damage and coronary artery disease [16–18]. Nevertheless, some studies have demonstrated that there was no significant relationship between the TyG index and carotid plaque, intracranial haemorrhage or cerebrovascular disease [10, 18, 19]. However, the relationship between the TyG index and cerebrovascular disease remains controversial. Therefore, we performed a systematic review and meta-analysis to investigate the relationship between the TyG index and cerebrovascular disease.

## Materials and methods

### Sources and methods of data retrieval

Our systematic review and meta-analysis was conducted according to the relevant PRISMA guidelines and extensions [20], and the PRISMA checklist is shown in the Supplemental Materials. The electronic databases, including PubMed, EMBASE, Cochrane Library, Web of Science and Medline, were searched from inception to March 2022 to explore the relationship between the TyG index and cerebrovascular disease. The following keywords (combined with the Boolean logical operator ‘OR.’ or ‘AND’) were used for the literature search: triglyceride glucose index, TyG index, triglyceride-glucose index, cerebrovascular disease, intracranial vascular disease, brain vascular disorder, stroke, brain ischemia, carotid artery disease, cerebral small vessel disease, brain vascular trauma, vascular dementia, intracranial arterial disorders and intracranial vasospasms. The literature search was restricted to articles published in English and articles with human subjects. The specific search strategies are listed in Tabl S1.

### Inclusion and exclusion criteria

**Fig. 1:**
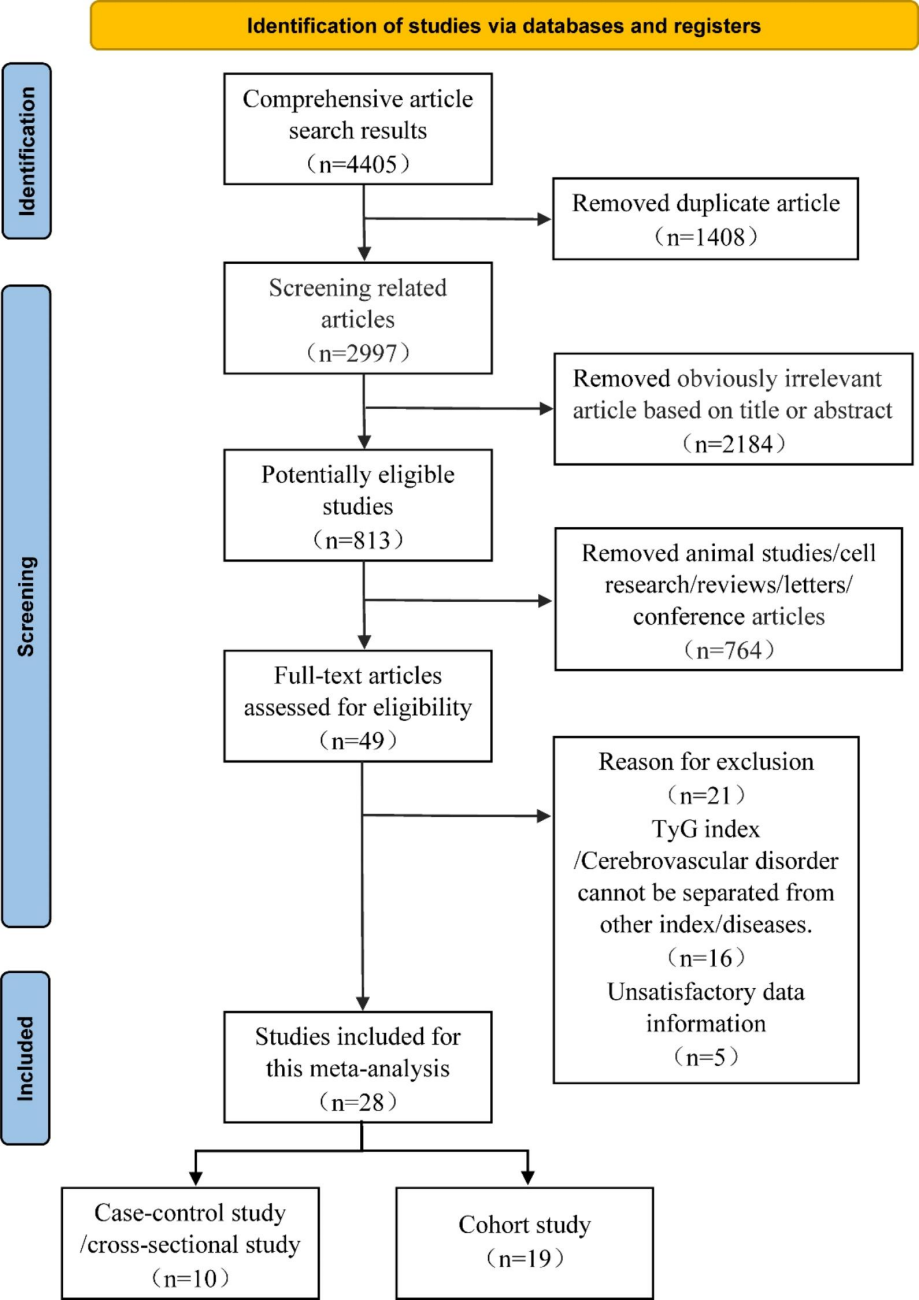
Flow diagram of the literature search and selection

The following criteria were used to identify the eligible articles: (1) the study design was an observational study; (2) the TyG index could be obtained via laboratory examinations and cerebrovascular disease must be an outcome disease; And (3) all of the outcomes are presented as odds ratios (ORs), relative risks (RRs) or hazard ratios (HRs), along with their corresponding 95% confidence intervals (CIs), for the relationship between the TyG index and cerebrovascular disease. Furthermore, we excluded some studies, such as in vitro studies, animal experiments, duplicate literature articles, reviews, letters or conference papers. Two researchers independently evaluated all of the relevant papers, extracted potentially eligible data and discussed and resolved disagreements with relevant experts (Fig. 1).

### Data abstraction

We extracted the following data from all of the included relevant studies: (1) the first author’s name, publication year, the nationality of the subjects, research design, number of participants, mean age and sex; (2) TyG index (mean ± standard deviation [SD]/median [interquartile range]) and types of cerebrovascular diseases; and (3) total effect estimates (OR, RR or HR), effect sizes of different subgroups (sex, age, body mass index, central obesity, diabetes, smoking, drinking, physical activity and hypertension) and quantiles and their corresponding 95% CIs.

### Quality assessment

The risk of bias for the observational literature was independently evaluated by two investigators by using the Newcastle‒Ottawa scale(NOS) [21], which included three parts (selection of the patients, comparability of the case/exposure groups and controls and exposure evaluation), and a study was awarded a maximum of one star for each numbered item within the selection and outcome categories. A maximum of two stars was given for comparability. Moreover, the Grading of Recommendations Assessment, Development and Evaluation (GRADE) system was used to classify the quality of evidence for the observational studies [22]. The included trials were classified as high quality, moderate quality, low quality or very low quality based on the risk of bias, inconsistency, indirectness, imprecision and publication bias.

### Statistical analysis

Statistical analyses were performed by using the statistical software RevMan version 5.3 and Stata version 13.0. The multivariate adjusted OR, RR and HR values from all of the individual studies were collected to calculate the pooled estimates and 95% CIs via the random effects model. Simultaneously, OR represented the case‒control study/cross‒sectional study, and RR/HR represented the cohort study. Cochran’s Q statistic and the *I*^*2*^ statistic were used to evaluate the statistical heterogeneity [23]. Significant heterogeneity was considered to be present if the *P* value was < 0.05, and we used the *I*^*2*^ value to estimate the degree of heterogeneity. *I*^*2*^ values of 25%, 50% and 75% indicate low, moderate and high levels of heterogeneity, respectively [24]. The sources of heterogeneity were explored via subgroup analyses and a sensitivity analysis. Subgroup analyses were conducted based on the region (Asia and Europe), basic illness, cerebrovascular disease (stroke, unclassified, vascular dementia, cerebral small vessel disease, carotid artery disease and intracranial arterial disease), sex of the subjects (male and female), age(< 60-years-old and ≥ 60-years-old), body mass index (normal and overweight/obese), central obesity, diabetes, smoking, drinking, physical activity and hypertension to evaluate the sources of heterogeneity.

The Egger’s test and a visual inspection of the funnel plots were used to estimate the potential publication bias [25]. The trim-and-fill method was conducted to evaluate the impact of bias on the outcomes [26]. Moreover, dose‒response analyses were performed to estimate the effect of every 1 mg/dl increase in the TyG index on cerebrovascular disease. In the dose‒response analysis, we used the median as the estimate for each interval and added or subtracted half of the difference between the medians of adjacent intervals as the estimate for the open interval.

## Results

In total, our study evaluated 4,405 relevant articles that were initially screened from electronic databases, but only 28 articles met our inclusion criteria, which contained a total of 11,944,688 participants. These 28 studies, involving nineteen cohort studies [10, 19, 27–43] and ten case‒control/cross‒sectional studies [16, 18, 38, 44–50] (with one of the articles including both cohort and cross‒sectional portions), investigated the risk of cerebrovascular disease in populations with different TyG index. The specific details are presented in Table 1. The risk of bias within the included studies was assessed via the NOS (Table 1, Tabl S2 and Table S3). Simultaneously, the GRADE system was utilized to classify the quality of the included evidence. The quality of the evidence in the cohort study was considered to be high (Table 2). In case‒control/cross‒sectional studies, the quality of the evidence was considered to be moderate because the dose‒response relationship remains unclear, due to limited number of studies (Table 3).

**Table 1:** Characteristics of the included observational studies

Study	Country	Design	Characteristics of participants	Number of participants	Mean age (years)	Male (%)	TyG index analysis	Outcomes reported	Variables adjusted	NOS
Hong S, et al.(1)	South Korea	Cohort	General population aged over 40 years	5,586,048	—	50.7	Categorized (Q4:Q1)	Vascular dementia	Age, sex, smoking status, alcohol consumption, physical activity, low income, BMI, hypertension and TC	8
Hong S, et al.(2)	South Korea	Cohort	General population aged over 40 years	5,593,134	—	50.5	Categorized (Q4:Q1)	Stroke	Age, sex, smoking, alcohol consumption, regular physical activity, low socioeconomic status, BMI, hypertension, TC, hypertension medications, warfarin and aspirin	8
Hu LL, et al.	China	Cohort	hypertension aged over 60 years	8487	68.8	52.8	Categorized (Q4:Q1); Continuous	Stroke	Age, sex, BMI, WC, education, physical activity, duration of hypertension, current smoking, current drinking, SBP, DBP, serum homocysteine, SUA, LDL-C, eGFR, diabetes mellitus, atrial fibrillation, CHD, anti-hypertensive drugs and anti-platelet drugs	8
Kim J, et al.	South Korea	Cohort	General population aged 40–79 years	144,603	—	54.0	Categorized (Q4:Q1)	Unclassified	Age, smoking status, drinking status, physical activity, BMI, SBP, LDL-C, economic status and anti-hypertensive medications	8
Alizargar J, et al.	China	Case control	General population aged over 30 years	276	56.2 ± 10.7	56.5	Continuous	Carotid artery disease	—	6
Laura S, et al.	Spain	Cohort	Internal medicine outpatient	5014	—	61.2	Categorized (Q5:Q1)	Unclassified	Age, sex, BMI, cigarette smoking, daily alcohol intake, lifestyle pattern, hypertension, type 2 diabetes, anti aggregation therapy, HDL-C, LDL-C	6
Li SS, et al.	China	Cohort	General population aged over 60 years	6078	70.5 ± 6.8	53.1	Categorized (Q4:Q1)	Unclassified	Age, sex, current smoking, alcohol consumption, exercise, BMI, resting heart rate, SBP, HDL-C, LDL-C, diabetic status	8
Liu Q, et al.	China	Cohort	General population	96,541	51.2 ± 12.6	79.6	Categorized (Q4:Q1); Continuous	Stroke	Age, sex, education, current smoking status, current drinking status, physical activity, BMI, hypertension, diabetes, HDL-C, LDL-C, hs-CRP, lipid-lowering medication, antidiabetic medication and antihypertensive medication	8
C. Irace, et al. (a)	Italy	Cross sectional	General population	330	—	56.7	Continuous	Carotid artery disease	—	5
C. Irace, et al. (b)	Italy	Cross sectional	General population	1432	—	57.6	Continuous	Carotid artery disease	—	5
Mao Q, et al.	China	Cohort	NSTE-ACS	438	62.5(53.0–68.0)	67.4	Categorized; cutoff: 8.805	Stroke	Age, gender, metabolic syndrome, LDL-C, HDL-C, SYNTAX score, CRP, basal insulin, sulfonylurea, metformin, α-glucosidase inhibitor, ACEI/ARB, beta-blocker and PCI/CABG	7
Nam Ki-W, et al.	South Korea	Cross sectional	General population aged 40–79 years	2615	56.0	53.0	Continuous	Cerebral small vessel disease	—	8
Shi WR, et al.	China	Case control	General population aged over 40 years	10,900	—	40.2	Categorized (Q4:Q1); Continuous	Stroke	Age, sex, education level, family annual income level, exercise level, current smoking, current drinking, WC, hypertension, BMI, HDL-C, LDL-C, history of cardiovascular diseases	7
Si S, et al.	England	Case control	General population aged 40–69 years	273,368	—	57.3	Categorized (Q4:Q1)	Unclassified	Age, sex, BMI, smoke status, fasting time and LDL-C	7
Wang AX, et al.(1)	China	Cohort	General population aged 18–98 years	62,443	49.1 ± 11.8	76.6	Categorized (Q4:Q1)	Stroke	Age, sex, TyG index at baseline, education, income, smoking status, drinking status, physical activity, BMI, SBP, DBP, history of hypertension, diabetes mellitus and dyslipidemia, antidiabetic agents, lipid-lowering agents, antihypertensive agents, HDL-C and hs-CRP at baseline	9
Wang AX, et al.(2)	China	Cohort	General population aged 18–98 years	97,653	51.7(43.5–59.0)	79.6	Categorized (Q4:Q1)	Stroke	Age, sex, level of education, income, smoking, alcohol abuse, physical activity, BMI, SBP, DBP, history of myocardial infarction, hypertension, diabetes mellitus, dyslipidemia, HDL-C, LDL-C, hs-CRP, antidiabetic drugs, lipid-lowering drugs and antihypertensive drugs	9
Wang AX, et al.(3)	China	Cross sectional	General population aged 18–98 years	4748	51.8(45.3–60.4)	58.9	Categorized (Q4:Q1); Continuous	Carotid artery disease	Age, sex, education, income, physical activity, smoking status, drinking status, history of hypertension and dyslipidemia, BMI, SBP, DBP, antihypertensive agents, lipid-lowering agents, HDL-C, LDL-C and hs-CRP	7
Wang AX, et al.(4 A)	China	Cross sectional	General population aged over 40 years	5381	52.5(45.6–61.6)	59.8	Categorized (Q4:Q1); Continuous	Intracranial arterial disease	Age, sex, BMI, education, income, physical activity, smoking status, drinking status, history of hypertension, diabetes, dyslipidemia, antihypertensive agents, antidiabetic agents, lipid-lowering agents, HDL-C, LDL-C and hs-CRP	8
Wang AX, et al.(4B)	China	Cohort	General population aged over 40 years	5381	52.5(45.6–61.6)	59.8	Categorized (Q4:Q1); Continuous	Intracranial arterial disease	Age, sex, BMI, education, income, physical activity, smoking status, drinking status, history of hypertension, diabetes, dyslipidemia, antihypertensive agents, antidiabetic agents lipid-lowering agents, HDL-C, LDL-C and hs-CRP	7
Chen L, et al.	China	Cohort	T2DM	1578	62.9 ± 8.0	70.7	Categorized (Q3:Q1)	Stroke	—	7
Chiu H, et al.	China	Cross sectional	T2DM	1990	—	43.0	Categorized (Q4:Q1)	Unclassified	—	5
Wang L, et al.	China	Cohort	diabetes and acute coronary syndrome	2531	66.3 ± 6.8	55.9	Categorized (Q3:Q1)	Stroke	Age, male, smoker, previous MI, previous CABG, BMI, LVEF, left main disease, multi-vessel disease, HbA1c, hs-CRP, statin and insulin	5
Wu ZY, et al.(a)	China	Cohort	General population aged over 18 years	6955	44.6 ± 10.1	61.3	Categorized (Q4:Q1); Continuous	Carotid artery disease	Age, sex, BMI, waist-hip ratio, hypertension, diabetes, dyslipidemia, SBP, fasting blood glucose, triglyceride, TC, SUA, eGFR, smoking status, drinking status, physical activity and IMT value at baseline	8
Wu ZY, et al.(b)	China	Cohort	General population aged over 18 years	8473	44.7 ± 9.8	56.8	Categorized (Q4:Q1); Continuous	Carotid artery disease	Age, sex, BMI, waist-hip ratio, hypertension, diabetes, dyslipidemia, SBP, fasting blood glucose, triglyceride, TC, SUA, eGFR, smoking status, drinking status, physical activity and IMT value at baseline	8
Zhang NN, et al.	China	Cross sectional	General population aged over 40 years	1938	—	47.7	Continuous	Intracranial arterial disease	Age, gender, hypertension, smoking habit, drinking habit, LDL-C, HDL-C, TC and obesity	8
Zhang Y, et al.(1)	China	Cohort	ACS	1655	—	73.9	Categorized; cutoff: 8.33	Stroke	—	5
Zhang Y, et al.(2)	China	Cohort	T2DM and acute MI	1932	65.4 ± 12.0	68.5	Categorized (Q3:Q1)	Stroke	Age, BMI, history of stroke and PCI, antiplatelet agent used before admission, WBC, hemoglobin, albumin, eGFR, LVEF, angiography findings, in-hospital treatment and hypoglycemic agents	7
Zhao Q, et al.	China	Cohort	NSTE-ACS without Diabetes	1510	59.7 ± 9.3	73.7	Continuous	Stroke	Age, gender, BMI, smoking history, hypertension, dyslipidemia, previous history of MI, PCI, stroke and PAD, diagnosis, TC, HDL-C, eGFR, HbA1c, LVEF, LM disease, three-vessel disease, chronic total occlusion, diffuse lesion, in-stent restenosis, SYNTAX score, treatment of LM, LCX, RCA, DES implantation, DCB application, complete revascularization, and number of stents, DAPT at admission, statins at admission, and ACEI/ARB at discharge	8
Guo QY, et al.	China	Cohort	prediabetes and ACS	2030	58.9 ± 10.3	74.1	Categorized; cutoff: 8.83	Stroke	—	5
Zhao S, et al.	China	Cross sectional	General population aged over 65 years	2830	71.5 ± 6.2	55.5	Categorized (Q4:Q1)	Carotid artery disease	Age, sex, BMI, WC, smoking habit, hypertension, family history of premature CVD, diabetes, HDL-C, LDL-C, insulin and statin therapy	7
Zhao Y, et al.	China	Cohort	General population aged over 40 years	11,777	—	40.9	Categorized (Q4:Q1)	Stroke	Age, gender, marital status, income, education level, smoking, alcohol drinking, physical activity, family history of stroke, SBP, DBP, resting heart rate, BMI, WC, TC, LDL-C and HDL-C	8

T2DM, Type 2 diabetes mellitus; ACS, Acute coronary syndrome; BMI, Body mass index; TC, total cholesterol; WC, waist circumference; SBP, systolic blood pressure; DBP, diastolic blood pressure; SUA, serum uric acid; LDL-C, low-density lipoprotein cholesterol; eGFR, estimated glomerular filtration rate; CHD, coronary heart disease; HDL-C, high-density lipoprotein cholesterol; hs-CRP, high sensitivity C-reactive protein; SYNTAX, synergy between PCI with taxus and cardiac surgery; CRP: C-reactive protein; ACEI, angiotensin converting enzyme inhibitors; ARB, angiotensin receptor blocker; PCI, percutaneous coronary intervention; CABG, coronary artery bypass grafting; MI, myocardial infarction; LVEF, left ventricular ejection fraction; TC total cholesterol; IMT, intima-media thickness; PCI, percutaneous coronary intervention; WBC, white blood cell; PAD, peripheral artery disease; LM, left main artery; LCX, left circumflex artery; RCA, right coronary artery; DES, drug-eluting stent; DCB, drug-coated balloon; DAPT, dual antiplatelet therapy; CVD, Cardiovascular diseases; TyG index, triglyceride glucose index; NOS, Newcastle-Ottawa scale;

**Table 2:** Summary of Findings (SoF) with the GRADE system (cohort studies)

High level of triglyceride glucose index compared with low level triglyceride glucose index in risk of cerebrovascular diseases.			
Population: Subjects with high level of triglyceride glucose index vs. low level triglyceride glucose index.			
Settings: Thirteen studies were conducted in Asia, one study were conducted in Europe.			
Cases: Subjects with high level of triglyceride glucose index.			
Controls: Subjects with low level of triglyceride glucose index.			
Outcomes	RR/HR (95% CI)^a^	No of participants (studies)	Quality of the evidence Comments (GRADE)
Risk of cerebrovascular diseases	1.22(1.14,1.30)	11,644,261 (19cohort studies)	⊕⊕⊕⊕ HIGH ^b,c^

GRADE working group grades of evidence

High quality: We are very confident that the true effect lies close to that of the estimate of the effect

Moderate quality: We are moderately confident in the effect estimate: The true effect is likely to be close to the estimate of the effect, but there is a possibility that it is substantially different

Low quality: Our confidence in the effect estimate is limited: The true effect may be substantially different from the estimate of the effect

Very low quality: We have very little confidence in the effect estimate: The true effect is likely to be substantially different from the estimate of effect

Abbreviations: CI, confidence interval; RR/HR, relative risk/hazard ratio

^a^ Results for cerebrovascular diseases risk of subjects with higher levels of triglyceride glucose index compared with lower triglyceride glucose index

^b^ Upgraded by one level due to all the results of the included studies were almost consistent (subjects with high triglyceride glucose index had high risk of cerebrovascular diseases)

^c^ Upgraded by one level due to a dose‒response relationship between cerebrovascular disease and triglyceride glucose index (The higher triglyceride glucose index, the higher risk of cerebrovascular diseases)

GRADE, Grading of Recommendations Assessment, Development and Evaluation system;

⊕, quality of evidence

**Table 3:** Summary of Findings (SoF) with the GRADE system (case‒control/cross‒sectional studies)

The level of triglyceride glucose index in people with cerebrovascular diseases compared with without cerebrovascular diseases.			
Population: Subjects with cerebrovascular diseases vs. normal subjects.			
Settings: Eight studies were conducted in Asia, two studies were conducted in Europe.			
Cases: Subjects with cerebrovascular diseases.			
Controls: normal subjects.			
Outcomes	OR (95% CI)^a^	No of participants (studies)	Quality of the evidence Comments (GRADE)
Risk of cerebrovascular diseases	1.15(1.07,1.23)	305,808 (10 case‒control /cross‒sectional studies)	⊕⊕⊕ MODERATE ^b^

GRADE working group grades of evidence

High quality: We are very confident that the true effect lies close to that of the estimate of the effect

Moderate quality: We are moderately confident in the effect estimate: The true effect is likely to be close to the estimate of the effect, but there is a possibility that it is substantially different

Low quality: Our confidence in the effect estimate is limited: The true effect may be substantially different from the estimate of the effect

Very low quality: We have very little confidence in the effect estimate: The true effect is likely to be substantially different from the estimate of effect

Abbreviations: CI, confidence interval; OR, odds ratio

^a^ Results for triglyceride glucose index levels of subjects with cerebrovascular diseases compared with controls

^b^ Upgraded by one level because triglyceride glucose index was associated with cerebrovascular diseases and all the results of the included studies were almost identical (subjects with cerebrovascular diseases had higher triglyceride glucose index)

GRADE, Grading of Recommendations Assessment, Development and Evaluation system;

⊕, quality of evidence

### Results of included cohort studies

**Fig. 2:**
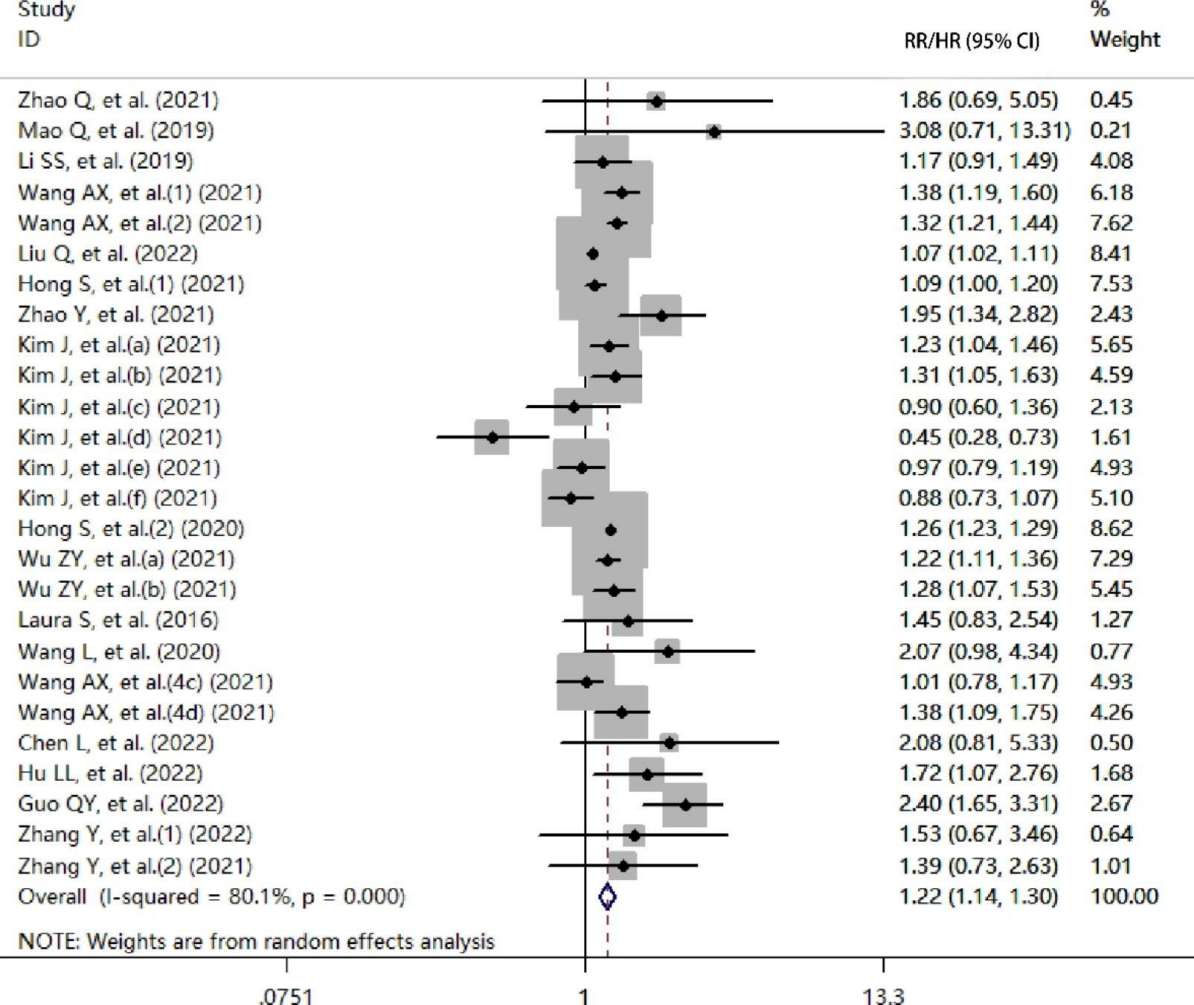
Forest plot of the risk of cerebrovascular disease in subjects with a high TyG index vs. a low TyG index (cohort studies; RR/HR, relative risk/hazard ratio; CI, confidence interval)

**Fig. 3:**
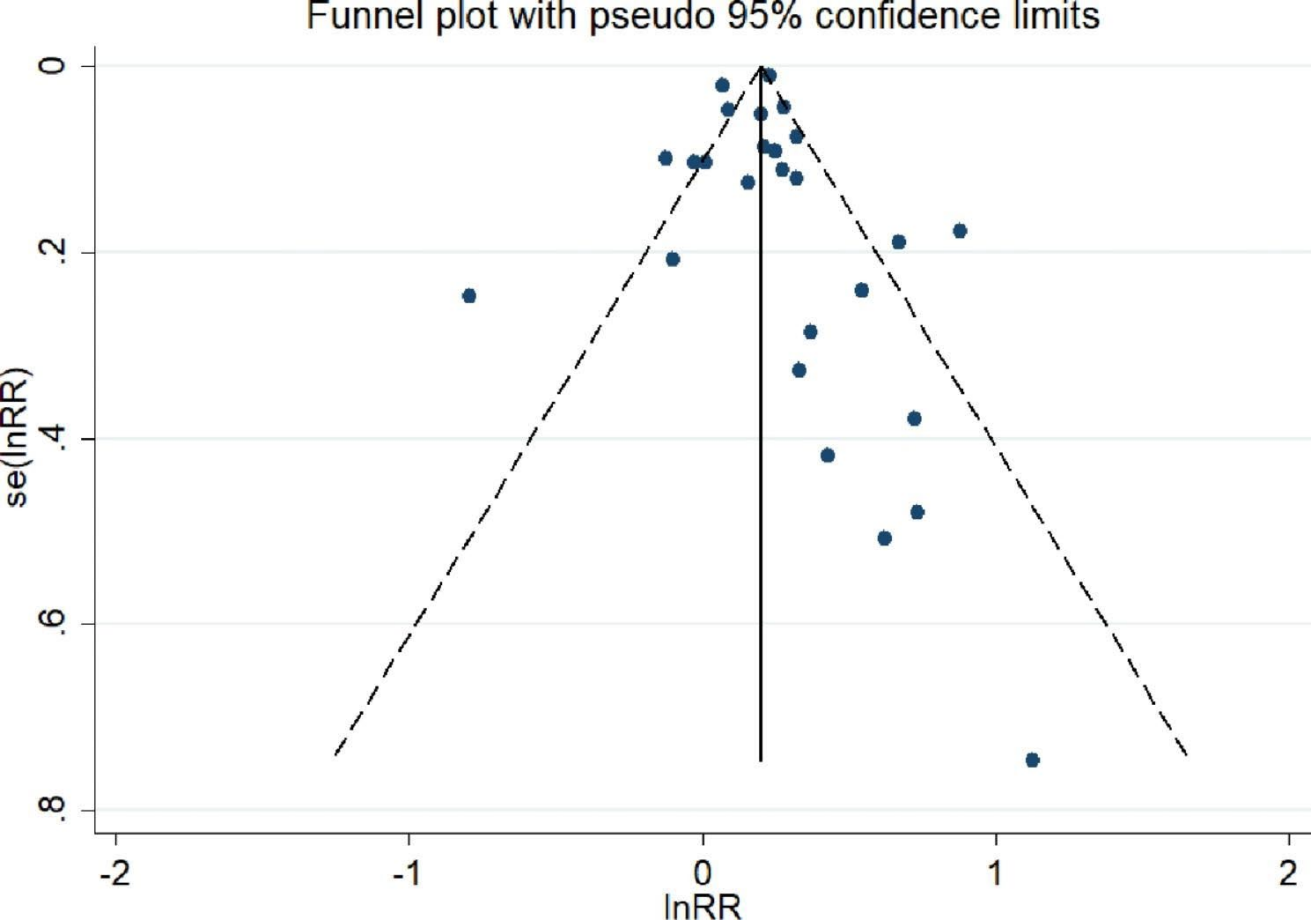
Funnel plot for the effect estimates of the TyG index (cohort studies; ln RR/HR = ln (relative risk/hazard ratio); se, standard error)

Nineteen cohort studies with 11,644,261 subjects were included in the study. The detailed characteristics of the participants are presented in Table 1. A higher TyG index increased the risk of cerebrovascular disease compared to a lower TyG index group (RR/HR = 1.22, 95% CI [1.14, 1.30], *P*<  0.001, Fig. 2). No publication bias was found via the Egger’s test and funnel plot (coefficient = 0.08, *t* = 0.14, *P* = 0.89, Fig. 3).

**Fig. 4:**
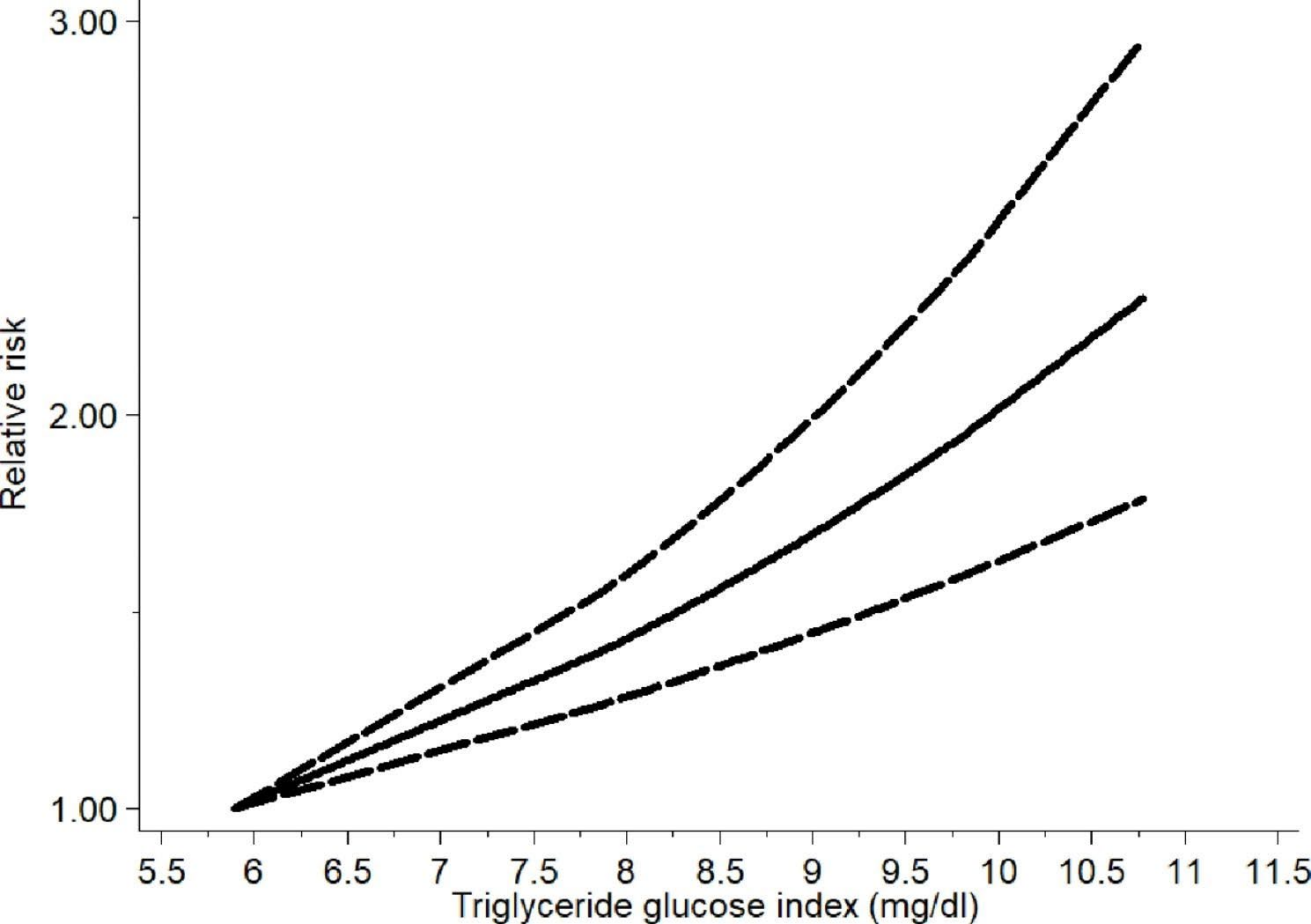
Dose-response plot of the TyG index and cerebrovascular disease (restricted cubic spline random effects model; bold dotted line, 95% confidence intervals for splines)

We analysed the source of heterogeneity via a sensitivity analysis and subgroup analyses. The sensitivity analysis showed no significant results; the details are presented in Figure S1. Furthermore, the subgroup analyses were performed in accordance with basic illness, cerebrovascular disease, region, sex of the subjects, age, diabetes, hypertension, smoking, drinking, physical activity, central obesity and body mass index. In subgroup analyses based on the type of cerebrovascular disease, the TyG index was related to stroke (RR/HR = 1.39, 95% CI [1.25, 1.55], *P*<  0.001). However, a similar relationship was not found in the unclassified group. Moreover, in the subgroup analyses of region, a higher TyG index increased the risk of cerebrovascular disease in the Asia group (RR/HR = 1.22, 95% CI [1.13,1.30], *P*<  0.001) but not in Europe. We also performed subgroup analyses based on age, sex and diabetes, and all of the results indicated that cerebrovascular disease was related to TyG index. The details of these results are shown in Table 4. Furthermore, the results of the dose‒response analysis demonstrated that a linear relationship was existed and the risk of cerebrovascular disease increased by 1.19 times per 1 mg/dl increment of the TyG index via a random-effects model (relative risk = 1.19, 95% CI [1.13,1.25], *P*<  0.001) (Fig. 4).

**Table 4:** Summary of results from the subgroup analyses of cohort studies

Subgrouped by	No. of studies	RR/HR (95%CI)	*I*^*2*^(%)	*P*_overall effect_	*P*_interaction_
Region	26	1.22(1.14,1.30)	80.1	< 0.001	0.54
Asia	25	1.22 (1.13,1.30)	80.8	< 0.001	
Europe	1	1.45(0.83,2.54)	—	0.19	
Basic illness	26	1.22(1.14,1.30)	80.1	< 0.001	< 0.001
no	18	1.17(1.09,1.25)	83.5	< 0.001	
yes	8	1.98(1.59,2.46)	0.0	< 0.001	
Cerebrovascular disease	26	1.22(1.14,1.30)	80.1	< 0.001	0.004
stroke	13	1.39(1.25,1.55)	84.9	< 0.001	
unclassified	8	1.03(0.86,1.22)	72.0	0.78	
vascular dementia	1	1.09(1.00,1.19)	—	0.06	
carotid artery disease	2	1.23(1.13,1.35)	0.0	< 0.001	
intracranial arterial disease	2	1.17(0.86,1.59)	74.0	0.31	
Sex	20	1.15(1.10,1.21)	69.7	< 0.001	0.78
male	10	1.17(1.12,1.22)	32.7	< 0.001	
female	10	1.15 (1.04,1.27)	81.4	0.007	
Age	12	1.17(1.11,1.24)	60.2	< 0.001	0.03
< 60	5	1.29(1.15,1.43)	39.0	< 0.001	
≥ 60	7	1.12(1.07,1.18)	51.6	< 0.001	
BMI	9	1.15(1.09,1.21)	84.2	< 0.001	0.002
normal	3	1.19(1.15,1.24)	59.9	< 0.001	
Overweight/obesity	6	1.09(1.05,1.14)	22.3	< 0.001	
Central obesity	4	1.12(1.09,1.16)	78.0	< 0.001	0.12
no	2	1.15(1.11,1.19)	64.0	< 0.001	
yes	2	1.10(1.06,1.14)	33.1	< 0.001	
Diabetes	16	1.20(1.14,1.28)	73.3	< 0.001	0.70
no	8	1.20(1.12,1.28)	80.2	< 0.001	
yes	8	1.23(1.12,1.35)	14.4	< 0.001	
Smoking	6	1.15(1.11,1.20)	77.9	< 0.001	0.58
no	3	1.18(1.08,1.28)	90.8	< 0.001	
yes	3	1.15(1.12,1.18)	0.0	< 0.001	
Drinking	6	1.15(1.10,1.21)	75.7	< 0.001	0.64
no	3	1.16(1.09,1.25)	88.8	< 0.001	
yes	3	1.14(1.06,1.22)	13.1	< 0.001	
Exercise	5	1.18(1.07,1.31)	69.1	0.001	0.08
no	1	1.10(1.05,1.15)	—	< 0.001	
yes	4	1.48(1.07,2.04)	75.4	0.02	
Blood pressure	6	1.16(1.09,1.22)	89.7	< 0.001	0.03
normal	3	1.20(1.13,1.27)	64.1	< 0.001	
hypertension	3	1.10(1.04,1.16)	63.1	< 0.001	

RR/HR, relative risk/hazard ratio; CI, confidence interval

### Results of included case-control/cross-sectional studies

**Fig. 5:**
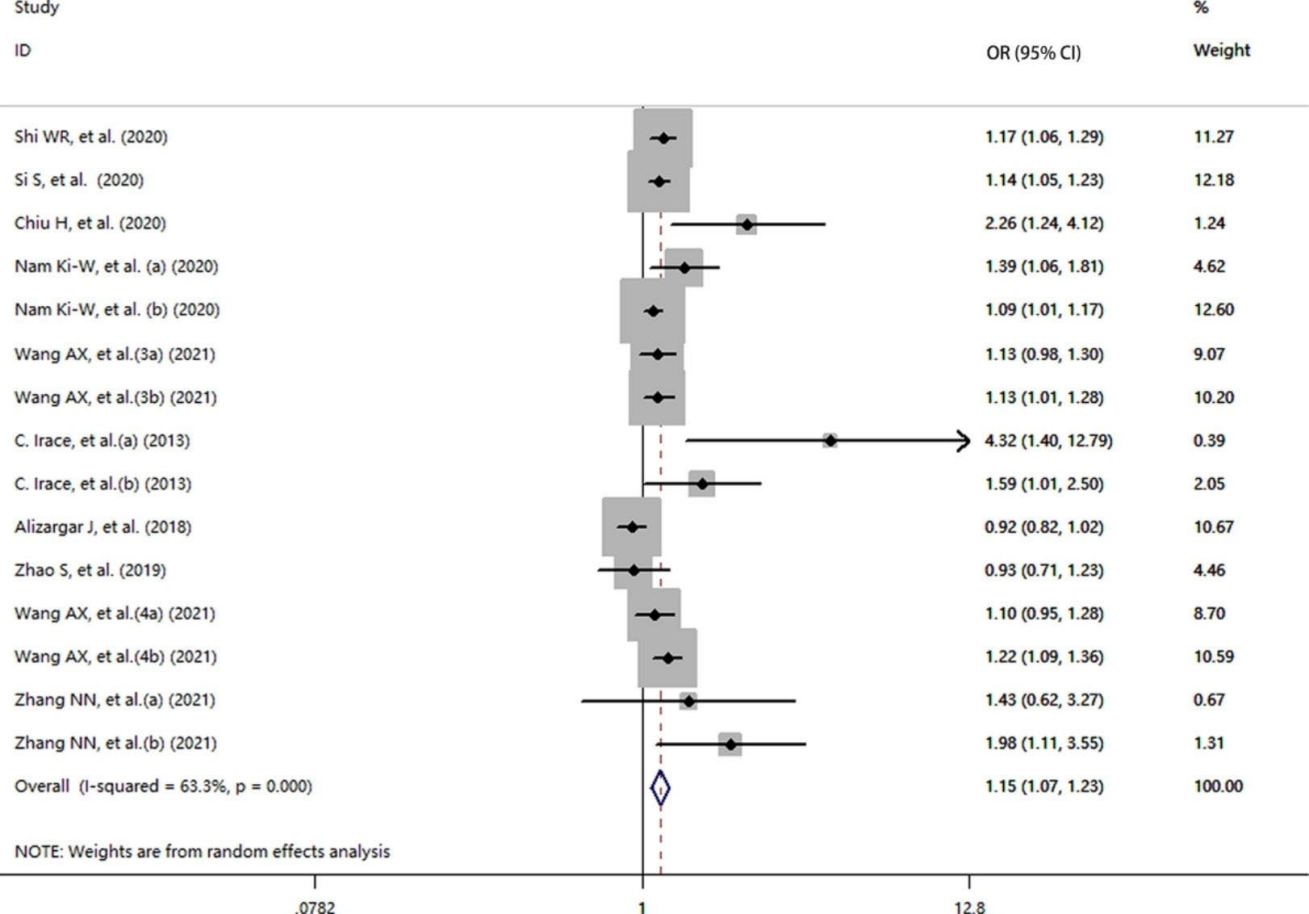
Forest plot of the risk of cerebrovascular disease in populations with a high TyG index vs. control groups (case-control/cross-sectional studies; OR, odds ratio; CI, confidence interval)

**Fig. 6:**
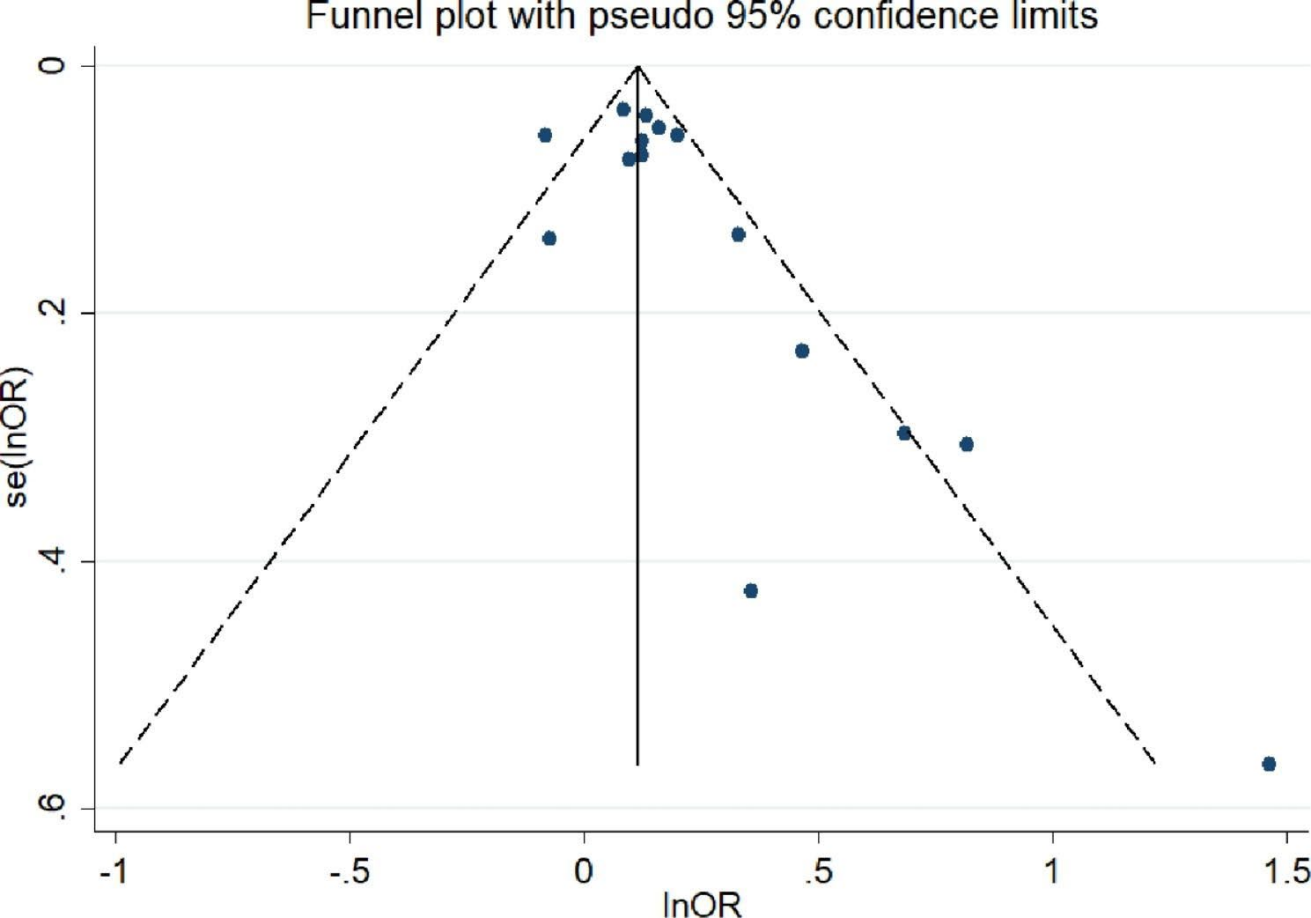
Funnel plot for the effect estimates of the TyG index (case-control/cross-sectional studies; lnOR = ln(odds ratio); se, standard error)

A total of 10 case‒control/cross‒sectional studies (including 305,808 samples) were included in our study, which investigated whether the risk of cerebrovascular disease was related to the TyG index. The detailed description and breakdown is shown in Table 1. The results indicated that the TyG index was higher in people with cerebrovascular disease. Moreover, the risk of cerebrovascular disease in the case group was 1.15 times that of the control group (OR = 1.15, 95% CI [1.07, 1.23], *P*<  0.001, Fig. 5). Furthermore, publication biases were found (coefficient = 1.58, *t* = 2.44, *P* = 0.03, Fig. 6). However, although the OR changed after the trim and fill method, the result was still statistically significant (adjusted: OR [95% CI]:1.113 [1.029,1.202], *P* = 0.007, number of trim and fill = 4), thus indicating that the publication bias had little effect on the results.

Similarly, a sensitivity analysis and subgroup analyses were used to identify the sources of heterogeneity. The sensitivity analysis demonstrated no significant results, and the details are shown in Figure S2. Additionally, we performed subgroup analyses based on the basic illness, cerebrovascular disease, region, sex of the subjects and diabetes. The results of the subgroup analysis when considering the type of cerebrovascular disease demonstrated that an association between the TyG index and intracranial arterial disease existed. (OR = 1.20, 95% CI [1.05,1.37], *P* = 0.006). However, the relationship was not observed in the other subgroups. Furthermore, the studies indicated that a high TyG index had a higher cerebrovascular disease risk than controls in the Asia subgroup (OR = 1.13, 95% CI [1.05,1.22], *P* = 0.001). The specific results are shown in Table 5.

**Table 5:** Summary of results from the subgroup analyses of case‒control/cross‒sectional studies

Subgrouped by	No. of studies	OR (95%CI)	*I*^*2*^(%)	*P*_overall effect_	*P*_interaction_
Region	15	1.15(1.07,1.23)	63.3	< 0.001	0.21
Asia	12	1.13(1.05,1.22)	63.0	0.001	
Europe	3	1.56(0.95,2.57)	73.4	0.08	
Basic illness	15	1.15(1.07,1.23)	63.3	< 0.001	0.02
no	14	1.13(1.06,1.21)	60.5	< 0.001	
yes	1	2.26(1.24,4.12)	—	0.008	
Cerebrovascular disease	15	1.15(1.07,1.23)	63.3	< 0.001	0.85
stroke	1	1.17(1.06,1.29)	—	0.001	
unclassified	2	1.50(0.78,2.91)	79.8	0.23	
cerebral small vessel disease	2	1.19(0.94,1.49)	66.8	0.14	
carotid artery disease	6	1.10(0.94,1.29)	72.9	0.24	
intracranial arterial disease	4	1.20(1.05,1.37)	31.8	0.006	
Sex	6	1.12(1.10,1.24)	44.4	0.03	0.39
male	3	1.08(0.98,1.19)	0.0	0.12	
female	3	1.23 (0.92,1.64)	77.0	0.16	
Diabetes	5	1.30(1.05,1.61)	34.7	0.02	0.04
no	2	1.18(1.03,1.36)	0.0	0.02	
yes	3	1.98(1.25,3.14)	0.0	0.004	

OR, odds ratio; CI, confidence interval

## Discussion

Cerebrovascular disease is one of the main causes of death and incapacity worldwide [1]. IR is an impaired tissue response to insulin stimulation, which ultimately leads to dysfunction in glucose and lipid metabolism [6]. Existing studies have proven that IR is intimately related to the occurrence of cerebrovascular disease or its triggers [6–10]. Recently, studies have suggested that the TyG index has potential as being an indicator of IR [14, 51]. However, it is uncertain as to whether a high TyG index increases the probability of developing cerebrovascular disease. Our meta-analysis found that the TyG index is related to cerebrovascular disease. Moreover, individuals with a high TyG index are more likely to develop cerebrovascular disease, and a potentially linear dose‒response relationship was observed.

Although the specific mechanism of action of the TyG index on cerebrovascular disease has not been elucidated, several potential mechanisms have been proposed that could be related to IR. First, IR activates inflammation-related genes [8]and interferes with insulin signalling at the level of intimal cells [52], thus resulting in varying degrees of oxidative responses, chronic inflammation and endothelial dysfunction [53, 54], which could impair vascular remodelling and growth and ultimately lead to cerebrovascular disease. Subsequently, IR induces proinflammatory and prothrombotic states by affecting platelet adhesion, activation and aggregation [55], thus resulting in endogenous fibrinolytic disturbances [56] and correspondingly triggering cerebrovascular lesions [57]. Finally, IR promotes foam cell formation at the onset of atherosclerosis and promotes late vulnerable plaque formation; in addition, in macrophages, it leads to plaque necrosis in advanced atherosclerosis by inducing prolonged endoplasmic reticulum stress and macrophage apoptosis [52, 58]. Additionally, IR can aggravate the effects of dyslipidaemia, diabetes, smoking and other factors on cerebrovascular disease and lead to the development of cerebrovascular disease [47]. Many studies have demonstrated that the TyG index is the indicator with the most potential for IR [14, 51]. Furthermore, the TyG index has also been proven to directly correlate with some traditional cerebrovascular risk factors and indicators, such as dyslipidaemia, diabetes, smoking, TG, LDL-C and hs-CRP [38, 59]. Therefore, given the important diagnostic value of the TyG index for IR and the reported direct association, we theorize that the level of the TyG index is closely related to cerebrovascular disease.

As the principal clinical type of cerebrovascular disease, stroke has elicited an increasing burden to the global health care system [3]. A subgroup analysis of our cohort study showed that subjects with a high TyG index had a 1.39-fold higher risk of developing stroke than those patients with a low TyG index, which was consistent with the studies of Zhao Y and Wang A et al. [10, 33]. Similar results were found in the carotid artery disease group. A possible mechanism has been previously mentioned. Furthermore, in the other subgroups, although a statistically significant difference was not found, a similar trend was observed.

In Asia, a high TyG index has been found to be associated with a higher probability of developing cerebrovascular disease than a low TyG index. Ethnicity could be a crucial factor affecting both cerebrovascular disease and the TyG index [60, 61]. Asian individuals can consume more carbohydrate-containing foods than Western people, which increases their blood triglyceride and glucose levels, thus increasing the likelihood of hypertriglyceridaemia and impaired fasting glucose [62, 63]. Insulin secretion may also be limited in Asian individuals relative to other regions [64]. Therefore, Asian individuals with a higher TyG index may be more prone to cardiovascular disease. Furthermore, in our meta-analysis, when considering the limited studies of other regions, more relevant future studies are needed.

Our research demonstrated that the risk of cerebrovascular disease in people younger than 60-years-old with a high TyG index was higher than that in people over 60-years-old. In recent years, it has been reported that incidences of obesity, hyperlipidaemia, hyperglycaemia, IR and other diseases have gradually been affecting younger individuals due to excessive intake of energy-intensive foods and a sedentary lifestyle [65, 66], which leads to higher levels of TyG in young people. The elderly population possesses more risk factors associated with cerebrovascular disease due to their increasing age, such as hypertension, metabolic disorders, degree of arterial stiffness and other vascular diseases [67, 68]. These factors may mask the influence of the TyG index on cerebrovascular disease. In contrast, in young people, the role of the TyG index on cerebrovascular disease was highlighted after excluding these risk factors. In addition, the administration of certain medications can also conceal this relationship [18].

This study had several limitations. For example, all of the studies that were included in this meta-analysis were observational studies, and the evidence level was lower. Moreover, a limited number of studies met our inclusion and exclusion criteria in this meta-analysis, and significant heterogeneity was discovered among them, which may be due to the presence of many confounding variables; therefore, a greater number of studies are needed to evaluate whether other study traits can influence the end results, such as participants’ ethnicities, diet, nutritional factors, dyslipidaemia, diabetes, clinical comorbidities, follow-up time and concomitant medications. Moreover, the sample sizes of the included studies were considerably different.

## Conclusion

In conclusion, our meta-analysis found that TyG index is related to cerebrovascular disease. When considering the limitations of this meta-analysis, more data and basic research are needed to verify the relationship between the TyG index and cerebrovascular disease.

## Electronic supplementary material

Below is the link to the electronic supplementary material.

Supplementary Material 1

Supplementary Material 2

Supplementary Material 3: Table S1: The specific search strategies

Supplementary Material 4: Table S2: The risk of bias for cohort studies by the Newcastle-Ottawa scale (NOS)

Supplementary Material 5: Table S3: The risk of bias for case-control/cross-sectional studies by the Newcastle-Ottawa scale (NOS)

Supplementary Material 6: PRISMA 2020 Checklist

## Data Availability

Not applicable.
