# Supplementary figures and images for: Association between triglyceride glucose index and risk of cerebrovascular disease: systematic review and meta-analysis
Source: Cardiovasc Diabetol. 2022 Nov 2;21:226. doi: 10.1186/s12933-022-01664-9 (PMC9632026; doi:10.1186/s12933-022-01664-9)

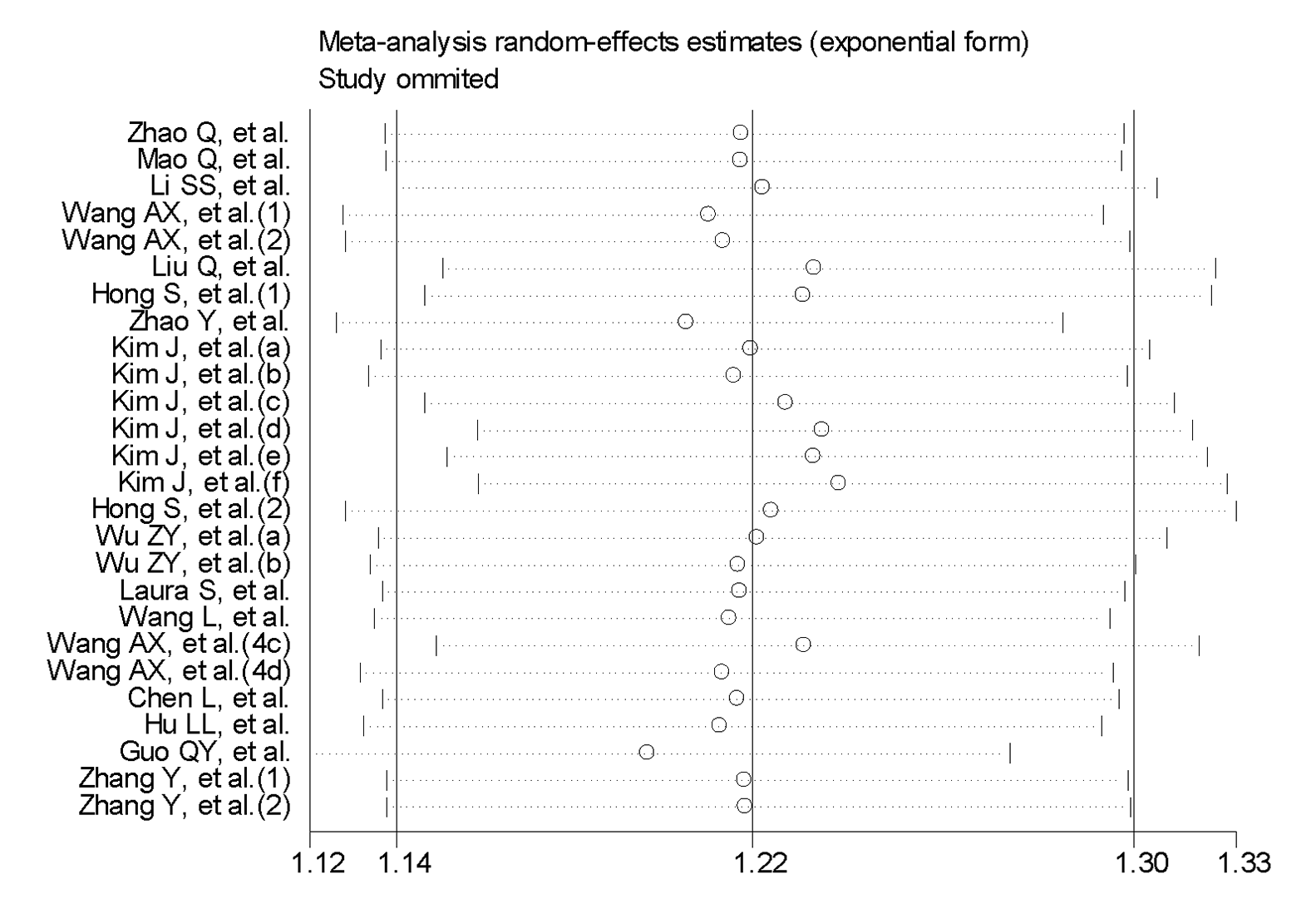

Supplement: Supplementary file 1 — Supplementary Material 1 [file 12933_2022_1664_MOESM1_ESM.png]

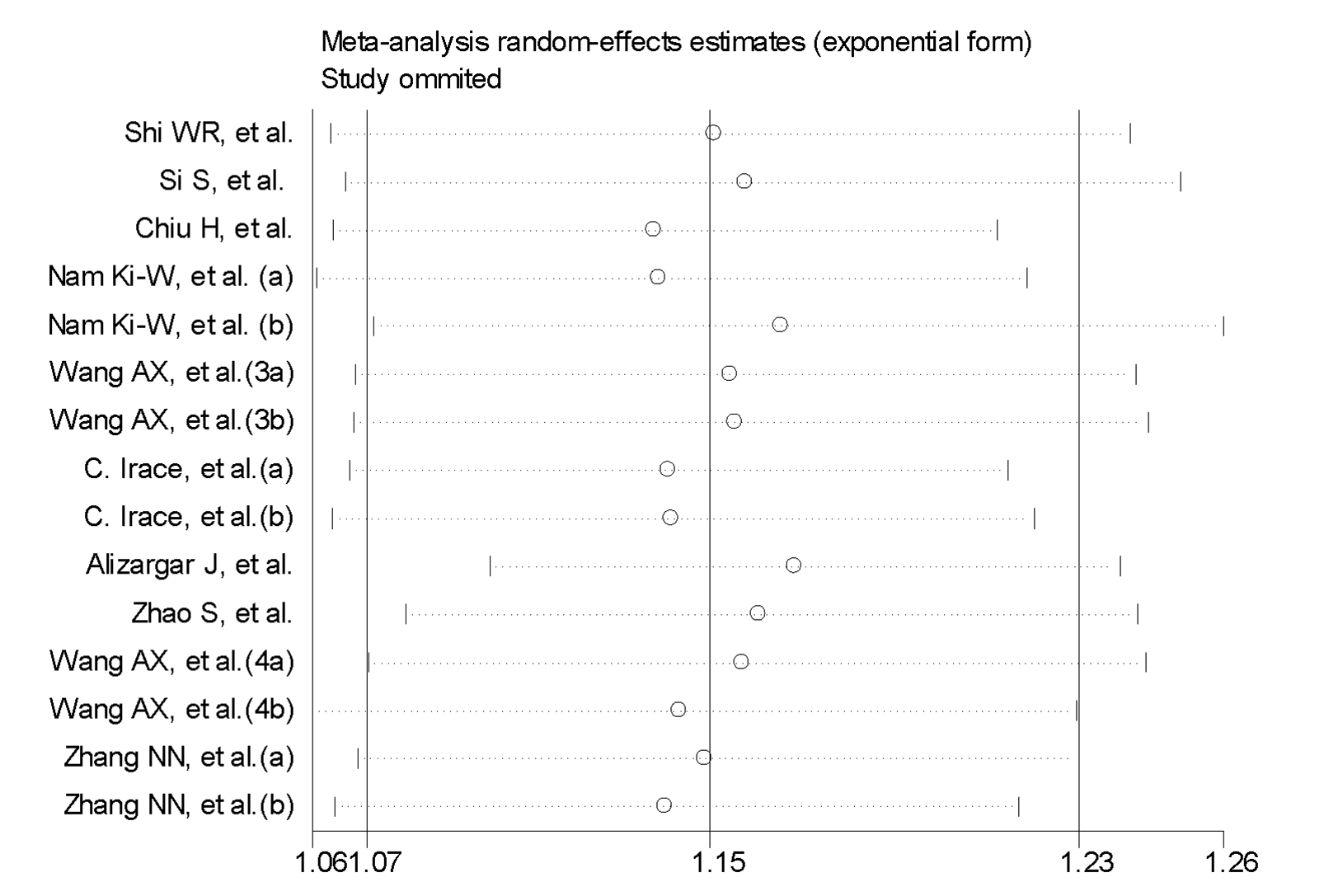

Supplement: Supplementary file 2 — Supplementary Material 2 [file 12933_2022_1664_MOESM2_ESM.png]
