# Supplementary material for: Association between triglyceride glucose index and risk of cerebrovascular disease: systematic review and meta-analysis
Source: Cardiovasc Diabetol. 2022 Nov 2;21:226. doi: 10.1186/s12933-022-01664-9 (PMC9632026; doi:10.1186/s12933-022-01664-9)
Supplement: Supplementary file 3 — Supplementary Material 3: Table S1: The specific search strategies [file 12933_2022_1664_MOESM3_ESM.docx]

**Table S1** The specific search strategies

| #1 | Cerebrovascular Disorder OR Intracranial Vascular Disease OR Intracranial Vascular Disorder OR Cerebrovascular Disease OR Brain Vascular Disorder OR Cerebrovascular Occlusion OR Cerebrovascular Insufficiency OR Cerebrovascular Insufficiencies OR CVD OR cerebrovascular accident |
| --- | --- |
| #2 | Basal Ganglia Cerebrovascular Disease OR Basal Ganglia Vascular Disease OR Lenticulostriate Vasculopathy OR Lenticulostriate Vasculopathies OR Lenticulostriate Vascular Disease OR Vascular Lenticulostriate Diseases OR Basal Ganglia Hemorrhage |
| #3 | Brain Ischemia OR Brain Ischemias OR Ischemic Encephalopathy OR Ischemic Encephalopathies OR Cerebral Ischemias OR Brain Infarction OR Brain Stem Infarctions OR Brain Ischemia Hypoxia OR TIA OR Transient Ischemic Attack OR Vertebrobasilar Insufficiency OR Subclavian Steal Syndrome |
| #4 | Carotid Artery Diseases OR Carotid Artery Disease OR Carotid Artery Disorders OR Carotid Artery Disorder OR Carotid Arterial Diseases OR Carotid Atherosclerosis OR Carotid Atheroscleroses OR Carotid Atherosclerotic Disease OR Internal Carotid Artery Diseases OR Common Carotid Artery Diseases OR External Carotid Artery Diseases OR Carotid Artery Injuries OR Traumatic Carotid Arteriopathy OR Carotid Artery Thrombosis OR Carotid Artery Thromboses OR Carotid-Cavernous Sinus Fistula OR C-C Fistula OR Carotid Stenosis OR Carotid Artery Narrowing OR Moyamoya Disease OR Progressive Intracranial Occlusive Arteropathy (Moyamoya) |
| #5 | Cerebral Small Vessel Diseases OR Cerebral Small Vessel Disease OR Cerebral Microangiopathies OR Cerebral Microangiopathy OR CADASIL OR Cerebral Autosomal Dominant Arteriopathy with Subcortical Infarcts and Leukoencephalopathy OR Cerebral Arteriopathy with Subcortical Infarcts and Leukoencephalopathy OR CADASILM OR Cerebral Amyloid Angiopathy, Familial OR HCHWA OR Autosomal Dominant Cerebrovascular Amyloidosis OR Fabry Disease OR alpha Galactosidase A Deficiency Disease OR Anderson-Fabry Disease OR MELAS Syndrome OR Mitochondrial Myopathy, Encephalopathy, Lactic Acidosis, And Stroke-Like Episodes OR Microscopic Polyangiitis OR Lacunar Stroke OR Lacunar Syndrome |
| #6 | Cerebrovascular Trauma OR Brain Vascular Trauma OR Brain Vascular Injury OR Vascular Brain Injuries OR Vascular Brain Injury OR Carotid Artery Injuries OR Traumatic Subarachnoid Hemorrhage OR Post Traumatic Subarachnoid Hemorrhage OR Vertebral Artery Dissection OR Dissecting Vertebral Artery Aneurysm |
| #7 | Vascular Dementia OR Acute Onset Vascular Dementia OR Subcortical Vascular Dementia OR Subcortical Vascular Dementias OR Arteriosclerotic Dementia OR Binswanger Disease OR Chronic Progressive Subcortical Encephalopathy OR Binswanger Encephalopathy OR Subcortical Leukoencephalopathies OR Binswanger's Diseas OR Subcortical Encephalopathy, Chronic Progressive OR Subcortical Leukoencephalopathy OR Subcortical Arteriosclerotic Encephalopathy OR Subcortical Arteriosclerotic Encephalopathies OR Binswanger's Encephalopathy OR CADASIL OR Multi-Infarct Dementias OR Dementia Multi-Infarct |
| #8 | Intracranial Arterial Disease OR Intracranial Arterial Disorders OR Intracranial Arterial Disorder OR Arterial Brain Disease OR Brain Arterial Disease OR Arterial Brain Disorder OR Arterial Brain Diseases OR Cerebral Arterial Diseases OR Intracranial Aneurysm OR Anterior Communicating Artery Aneurysm OR Intracranial Arteriosclerosis OR Cerebral Arterioscleroses |
| #9 | Intracranial Arteriovenous Malformations OR Intracranial Arteriovenous Malformation OR Congenital Intracranial Arteriovenous Malformations OR AVM (Arteriovenous Malformation) Intracranial OR Ruptured Intracranial Arteriovenous Malformation OR Cerebral Arteriovenous Malformation OR Cerebral Arteriovenous Malformations OR Vein of Galen Malformations |
| #10 | Intracranial Embolism and Thrombosis OR Cerebral Embolism and Thrombosis OR Brain Embolism and Thrombosis OR Carotid Artery Thrombosis OR Intracranial Embolism OR Brain Embolus OR Intracranial Thrombosis OR Cerebral Thromboses |
| #11 | Intracranial Hemorrhages OR Intracranial Hemorrhage OR Posterior Fossa Hemorrhage OR Posterior Fossa Hemorrhages OR Brain Hemorrhage OR Brain Hemorrhages OR Cerebral Hemorrhage OR Cerebral Parenchymal Hemorrhage OR Hypertensive Intracranial Hemorrhage OR Traumatic Intracranial Hemorrhage OR Pituitary Apoplexy OR Subarachnoid Hemorrhage OR SAH OR Spontaneous Subarachnoid Hemorrhage |
| #12 | Periventricular Leukomalacia OR Periventricular Leukomalacias OR Periventricular Encephalomalacia OR Neonatal Cerebral Leukomalacia OR Neonatal Cerebral Leukomalacias OR Cystic Periventricular Leukomalacia OR White Matter OR white matter |
| #13 | Sneddon Syndrome OR Livedo Reticularis And Cerebrovascular Accidents |
| #14 | Stroke OR Cerebrovascular Accident OR Cerebrovascular Accidents OR CVA OR CVAs OR Cerebrovascular Apoplexy OR Brain Vascular Accident OR Cerebrovascular Stroke OR Apoplexy OR Cerebral Stroke OR Cerebral Strokes OR Acute Stroke OR Acute Cerebrovascular Accident OR Acute Cerebrovascular Accidents OR Brain Infarction OR Hemorrhagic Strokes OR Subarachnoid Hemorrhagic Stroke OR Intracerebral Hemorrhagic Stroke OR Intracerebral Hemorrhage Stroke OR Intracerebral Hemorrhage Strokes OR Ischemic Strokes OR Ischaemic Stroke OR Cryptogenic Ischemic Stroke OR Cryptogenic Ischemic Strokes OR Cryptogenic Stroke OR Cryptogenic Embolism Stroke OR Wake-up Stroke OR Wake up Stroke OR Wake-up Strokes OR Acute Ischemic Stroke OR Embolic Stroke OR Thrombotic Stroke |
| #15 | Susac Syndrome OR Retinocochleocerebral Vasculopathy |
| #16 | Vascular Headache OR Vascular Cephalgia |
| #17 | CNS Vasculitis OR Central Nervous System Angiitis OR Central Nervous System Vasculitis OR Secondary CNS Vasculitis OR Cerebral Angiitis OR Cerebral Vasculitis OR Granulomatous Angiitis OR Granulomatous Arteritis OR Central Nervous System AIDS Arteritis OR HIV Associated Vasculitis of the Central Nervous System OR Giant Cell Arteritides OR Horton's Giant Cell Arteritis OR Central Nervous System Lupus Vasculitis |
| #18 | Intracranial Vasospasms OR Intracranial Vasospasm OR Intracranial Vascular Spasm OR Intracranial Angiospasm OR Cerebral Vasospasm OR Cerebrovascular Spasm OR Cerebral Angiospasm OR Cerebral Artery Spasm |
| #19 | #1 OR #2 OR #3 OR #4 OR #5 OR #6 OR #7 OR #8 OR #9 OR #10 OR #11 OR #12 OR #13 OR #14 OR #15 OR #16 OR #17 OR #18 |
| #20 | triglyceride-glucose index OR triglyceride glucose index OR TyG index OR triglyceride and glucose index OR triglyceride–glucose (T/Gly) index OR TyGs OR triglyceride glucose indices OR The triglyceride-glucose index OR Triglyceride/glucose index OR Triglycerides and glucose index |
| #21 | #19 AND #20 |
