# Supplementary material for: Association between triglyceride glucose index and risk of cerebrovascular disease: systematic review and meta-analysis
Source: Cardiovasc Diabetol. 2022 Nov 2;21:226. doi: 10.1186/s12933-022-01664-9 (PMC9632026; doi:10.1186/s12933-022-01664-9)
Supplement: Supplementary file 4 — Supplementary Material 4: Table S2: The risk of bias for cohort studies by the Newcastle-Ottawa scale (NOS) [file 12933_2022_1664_MOESM4_ESM.docx]

**Table S2** The risk of bias for cohort studies by the Newcastle-Ottawa scale (NOS)

**Zhao Q, et al. 2021**

| Study type | Cohort study | | |
| --- | --- | --- | --- |
| Participants | Patients without diabetes who were diagnosed with non-ST-segment elevation acute coronary syndrome and treated with elective percutaneous coronary intervention.  Sample size: 1510  Mean age in years: 59.7±9.3  Gender: 1113males/397females  Location: China | | |
| Outcomes | Main study outcome: the predictive value of the triglyceride glucose index for adverse cardiovascular events, triglyceride glucose index and relationships to demographics.  Available outcomes: different quantile triglyceride glucose index and incidence of cardiovascular diseases(nonfatal ischemic stroke). | | |
| ***Risk of bias*** | | | |
| **Bias** | | **Authors’ judgment** | **Support for judgment** |
| Representativeness of the exposed cohort (**Selection**) | | 1 | truly representative of the average nondiabetic patients diagnosed with non-ST-segment elevation acute coronary syndrome and received elective percutaneous coronary intervention in the community |
| Selection of the non exposed cohort (**Selection**) | | 1 | drawn from the same community as the exposed cohort |
| Ascertainment of exposure (**Selection**) | | 1 | secure record (eg laboratory examination) |
| Demonstration that outcome of interest was not present at start of study (**Selection**) | | 1 | some people have a history of stroke, but no statistical difference (endpoint). |
| Comparability of cohorts on the basis of the design or analysis (**Comparability**) | | 2 | study controls for population, age, body mass index and other factors |
| Assessment of outcome (**Outcome**) | | 1 | independent blind assessment(medical records) |
| Was follow up long enough for outcomes to occur (**Outcome**) | | 1 | yes(48 months) |
| Adequacy of follow up of cohorts (**Outcome**) | | 0 | no statement |

**Mao Q, et al. 2019**

| Study type | Cohort study | | |
| --- | --- | --- | --- |
| Participants | Patients diagnosed with non-ST-segment elevation acute coronary syndrome  Sample size: 438  Median age (IQR): 62.5 (53.0–68.0)  Gender: 295males/143females  Location: China | | |
| Outcomes | Main study outcome: the association of the TyG index with cardiovascular risk factors and outcomes, triglyceride glucose index and relationships to demographics.  Available outcomes: different level triglyceride glucose index and incidence of cardiovascular diseases (nonfatal stroke). | | |
| ***Risk of bias*** | | | |
| **Bias** | | **Authors’ judgment** | **Support for judgment** |
| Representativeness of the exposed cohort (**Selection**) | | 1 | somewhat representative of the average non-ST-segment elevation acute coronary syndrome in the community |
| Selection of the non exposed cohort (**Selection**) | | 1 | drawn from the same community as the exposed cohort |
| Ascertainment of exposure (**Selection**) | | 1 | secure record (eg laboratory examination) |
| Demonstration that outcome of interest was not present at start of study (**Selection**) | | 0 | some people have a history of stroke (endpoint) |
| Comparability of cohorts on the basis of the design or analysis (**Comparability**) | | 2 | study controls for population, age, body mass index and other factors |
| Assessment of outcome (**Outcome**) | | 1 | record linkage (hospital records or by interviewing patients and their families) |
| Was follow up long enough for outcomes to occur (**Outcome**) | | 1 | yes(12 months) |
| Adequacy of follow up of cohorts (**Outcome**) | | 0 | no statement |

**Li SS, et al. 2019**

| Study type | Cohort study | | |
| --- | --- | --- | --- |
| Participants | Participants of 60 years of age or older who entered the health check-up program  Sample size: 6078  Mean age in years: 70.45±6.79  Gender: 3226males/2852females  Location: China | | |
| Outcomes | Main study outcome: the role of the triglyceride glucose index in the development of cardiovascular events.  Available outcomes: different level triglyceride glucose index and incidence of cardiovascular diseases (cerebrovascular diseases). | | |
| ***Risk of bias*** | | | |
| **Bias** | | **Authors’ judgment** | **Support for judgment** |
| Representativeness of the exposed cohort **(Selection)** | | 1 | truly representative of the average in Xinzheng and Xinmi City in the community |
| Selection of the non exposed cohort **(Selection)** | | 1 | drawn from the same community as the exposed cohort |
| Ascertainment of exposure **(Selection)** | | 1 | secure record (eg laboratory examination) |
| Demonstration that outcome of interest was not present at start of study **(Selection)** | | 1 | yes (endpoint) |
| Comparability of cohorts on the basis of the design or analysis **(Comparability)** | | 2 | study controls for age, body mass index and other factors |
| Assessment of outcome **(Outcome)** | | 1 | the data of the annual health check-up program with a digital linkage to the hospital dataset for admissions |
| Was follow up long enough for outcomes to occur **(Outcome)** | | 0 | no (no description) |
| Adequacy of follow up of cohorts **(Outcome)** | | 1 | subjects lost to follow up unlikely to introduce bias, description of those lost |

**Wang AX, et al. (1) 2021**

| Study type | Cohort study | | |
| --- | --- | --- | --- |
| Participants | The general population aged 18-90  Sample size: 62443  Mean age in years: 49.07±11.84  Gender: 47827 males/14616females  Location: China | | |
| Outcomes | Main study outcome: change in triglyceride-glucose index predicts the risk of cardiovascular diseases.  Available outcomes: different level triglyceride glucose index and incidence of cardiovascular diseases (stroke). | | |
| ***Risk of bias*** | | | |
| **Bias** | | **Authors’ judgment** | **Support for judgment** |
| Representativeness of the exposed cohort **(Selection)** | | 1 | truly representative of the average in Tangshan City in the community |
| Selection of the non exposed cohort **(Selection)** | | 1 | drawn from the same community as the exposed cohort |
| Ascertainment of exposure **(Selection)** | | 1 | secure record (eg health examination) |
| Demonstration that outcome of interest was not present at start of study **(Selection)** | | 1 | yes (excluded people with stroke) |
| Comparability of cohorts on the basis of the design or analysis **(Comparability)** | | 2 | study controls for age, sex and other factors |
| Assessment of outcome **(Outcome)** | | 1 | independent blind assessment according the World Health Organization criteria |
| Was follow up long enough for outcomes to occur **(Outcome)** | | 1 | yes(10years) |
| Adequacy of follow up of cohorts **(Outcome)** | | 1 | subjects lost to follow up unlikely to introduce bias ,description of those lost |

**Wang AX, et al. (2) 2021**

| Study type | Cohort study | | |
| --- | --- | --- | --- |
| Participants | The general population aged 18-98  Sample size: 97653  Median age (IQR): 51.67 (43.53–58.97)  Gender: 77748 males/19905females  Location: China | | |
| Outcomes | Main study outcome: triglyceride glucose index and the risk of stroke and its subtypes.  Available outcomes: different level triglyceride glucose index and incidence of stroke. | | |
| ***Risk of bias*** | | | |
| **Bias** | | **Authors’ judgment** | **Support for judgment** |
| Representativeness of the exposed cohort **(Selection)** | | 1 | truly representative of the average in Tangshan City in the community |
| Selection of the non exposed cohort **(Selection)** | | 1 | drawn from the same community as the exposed cohort |
| Ascertainment of exposure **(Selection)** | | 1 | secure record (eg health examination) |
| Demonstration that outcome of interest was not present at start of study **(Selection)** | | 1 | yes (excluded people with stroke) |
| Comparability of cohorts on the basis of the design or analysis **(Comparability)** | | 2 | study controls for age, sex and other factors |
| Assessment of outcome **(Outcome)** | | 1 | independent blind assessment according the World Health Organization criteria |
| Was follow up long enough for outcomes to occur **(Outcome)** | | 1 | yes (11years) |
| Adequacy of follow up of cohorts **(Outcome)** | | 1 | subjects lost to follow up unlikely to introduce bias ,description of those lost |

**Liu Q, et al. 2020**

| Study type | Cohort study | | |
| --- | --- | --- | --- |
| Participants | General population.  Sample size: 96541  Mean age in years: 51.19 ± 12.57  Gender: 76858 males/19683females  Location: China | | |
| Outcomes | Main study outcome: whether triglyceride glucose index is an independent risk factor for cardiovascular diseases.  Available outcomes: different level triglyceride glucose index and incidence of stroke. | | |
| ***Risk of bias*** | | | |
| **Bias** | | **Authors’ judgment** | **Support for judgment** |
| Representativeness of the exposed cohort **(Selection)** | | 1 | truly representative of the average in Tangshan City in the community |
| Selection of the non exposed cohort **(Selection)** | | 1 | drawn from the same community as the exposed cohort |
| Ascertainment of exposure **(Selection)** | | 1 | secure record (eg laboratory examination) |
| Demonstration that outcome of interest was not present at start of study **(Selection)** | | 1 | yes (excluded people with history of cardiovascular diseases) |
| Comparability of cohorts on the basis of the design or analysis **(Comparability)** | | 2 | study controls for population, body mass index and other factors |
| Assessment of outcome **(Outcome)** | | 1 | independent blind assessment according the World Health Organization criteria |
| Was follow up long enough for outcomes to occur **(Outcome)** | | 1 | yes (10years) |
| Adequacy of follow up of cohorts **(Outcome)** | | 0 | no statement |

**Hong S, et al. (1) 2021**

| Study type | Cohort study | | |
| --- | --- | --- | --- |
| Participants | Participants who are 40 years of age or older participating in the National Health Screening Program  Sample size: 5586048  Age: NA  Gender: 2831762 males/ 2754286 females  Location: South Korea | | |
| Outcomes | Main study outcome: the potential relationships between the triglyceride glucose index and dementia.  Available outcomes: different level triglyceride glucose index and incidence of vascular dementia. | | |
| ***Risk of bias*** | | | |
| **Bias** | | **Authors’ judgment** | **Support for judgment** |
| Representativeness of the exposed cohort **(Selection)** | | 1 | truly representative of the average in Korea in the community |
| Selection of the non exposed cohort **(Selection)** | | 1 | drawn from the same community as the exposed cohort |
| Ascertainment of exposure **(Selection)** | | 1 | secure record (eg laboratory examination) |
| Demonstration that outcome of interest was not present at start of study **(Selection)** | | 1 | yes (excluded people with history of dementia) |
| Comparability of cohorts on the basis of the design or analysis **(Comparability)** | | 2 | study controls for population, age and other factors |
| Assessment of outcome **(Outcome)** | | 1 | ICD-10 diagnosis codes for dementia |
| Was follow up long enough for outcomes to occur **(Outcome)** | | 1 | yes (8years) |
| Adequacy of follow up of cohorts **(Outcome)** | | 0 | no statement |

**Zhao Y, et al. 2021**

| Study type | Cohort study | | |
| --- | --- | --- | --- |
| Participants | Rural population over 40  Sample size: 11777  Age: NA  Gender: 4815males/6962females  Location: China | | |
| Outcomes | Main study outcome: the association between triglyceride-glucose and incident ischemic stroke, the potential effect of modification by several known risk factors of stroke.  Available outcomes: different level triglyceride glucose index and incidence of ischemic stroke. | | |
| ***Risk of bias*** | | | |
| **Bias** | | **Authors’ judgment** | **Support for judgment** |
| Representativeness of the exposed cohort **(Selection)** | | 0 | truly representative of the average the rural Chinese in the community |
| Selection of the non exposed cohort **(Selection)** | | 1 | drawn from the same community as the exposed cohort |
| Ascertainment of exposure **(Selection)** | | 1 | secure record (eg laboratory examination) |
| Demonstration that outcome of interest was not present at start of study **(Selection)** | | 1 | yes (free of stroke) |
| Comparability of cohorts on the basis of the design or analysis **(Comparability)** | | 2 | study controls for population, age and other factors |
| Assessment of outcome **(Outcome)** | | 1 | clearly defined |
| Was follow up long enough for outcomes to occur **(Outcome)** | | 1 | yes (8years) |
| Adequacy of follow up of cohorts **(Outcome)** | | 1 | subjects lost to follow up unlikely to introduce bias (select an adequate 85.5% follow up) |

**Kim J, et al. 2021**

| Study type | Cohort study | | |
| --- | --- | --- | --- |
| Participants | Subjects 40-79 years old  Sample size: 144603  Age: NA  Gender:78021males/66582females  Location: South Korea | | |
| Outcomes | Main study outcome: the association between triglyceride-glucose index, cardio-cerebrovascular diseases, and mortality.  Available outcomes: different level triglyceride glucose index and incidence of cardio-cerebrovascular diseases (Ischemic \ Hemorrhagic\ Other cerebrovascular disease). | | |
| ***Risk of bias*** | | | |
| **Bias** | | **Authors’ judgment** | **Support for judgment** |
| Representativeness of the exposed cohort (Selection) | | 1 | truly representative of the average Korean in the community |
| Selection of the non exposed cohort **(Selection)** | | 1 | drawn from the same community as the exposed cohort |
| Ascertainment of exposure **(Selection)** | | 1 | secure record (eg laboratory examination) |
| Demonstration that outcome of interest was not present at start of study **(Selection)** | | 1 | yes (excluded people with history of cardio-cerebrovascular diseases) |
| Comparability of cohorts on the basis of the design or analysis **(Comparability)** | | 2 | study controls for population(diabetes, medical history), age and other factors |
| Assessment of outcome **(Outcome)** | | 1 | clearly defined |
| Was follow up long enough for outcomes to occur **(Outcome)** | | 1 | yes (8years) |
| Adequacy of follow up of cohorts **(Outcome)** | | 0 | no statement |

**Hong S, et al. (2) 2020**

| Study type | Cohort study | | |
| --- | --- | --- | --- |
| Participants | Participants who are 40 years of age or older participating in the National Health Screening Program  Sample size: 5593134  Age: NA  Gender:2826348males/2766786females  Location: South Korea | | |
| Outcomes | Main study outcome: the relationship between triglyceride glucose index and cardiovascular diseases.  Available outcomes: different level triglyceride glucose index and incidence of cardiovascular diseases (stroke). | | |
| ***Risk of bias*** | | | |
| **Bias** | | **Authors’ judgment** | **Support for judgment** |
| Representativeness of the exposed cohort **(Selection)** | | 1 | truly representative of the average South Korea in the community |
| Selection of the non exposed cohort **(Selection)** | | 1 | drawn from the same community as the exposed cohort |
| Ascertainment of exposure **(Selection)** | | 1 | secure record (eg laboratory examination) |
| Demonstration that outcome of interest was not present at start of study **(Selection)** | | 1 | yes (excluded people with history of atherosclerotic cardiovascular diseases) |
| Comparability of cohorts on the basis of the design or analysis **(Comparability)** | | 2 | study controls for population (no diabetes and no hyperlipidemia), age, body mass index and other factors |
| Assessment of outcome **(Outcome)** | | 1 | clearly defined |
| Was follow up long enough for outcomes to occur **(Outcome)** | | 1 | yes (15 years) |
| Adequacy of follow up of cohorts **(Outcome)** | | 0 | no statement |

**Wu ZY, et al. (a) 2021**

| Study type | Cohort study | | |
| --- | --- | --- | --- |
| Participants | Adults undergoing annual health examinations.  Sample size: 6955  Mean age in years: 44.56±10.11  Gender: 4264 males/2691females  Location: China | | |
| Outcomes | Main study outcome: investigate whether the triglyceride glucose index increases the risk of carotid atherosclerosis incidence.  Available outcomes: different level triglyceride glucose index and incidence of carotid atherosclerosis. | | |
| ***Risk of bias*** | | | |
| **Bias** | | **Authors’ judgment** | **Support for judgment** |
| Representativeness of the exposed cohort (Selection) | | 1 | truly representative of the average Beijing in the community |
| Selection of the non exposed cohort **(Selection)** | | 1 | drawn from the same community as the exposed cohort |
| Ascertainment of exposure **(Selection)** | | 1 | secure record (eg laboratory examination) |
| Demonstration that outcome of interest was not present at start of study **(Selection)** | | 1 | yes (excluded participants with carotid plaques) |
| Comparability of cohorts on the basis of the design or analysis **(Comparability)** | | 2 | study controls for population(no coronary heart disease, stroke or malignant tumor), age, and other factors |
| Assessment of outcome **(Outcome)** | | 1 | clearly defined |
| Was follow up long enough for outcomes to occur **(Outcome)** | | 1 | yes (7years) |
| Adequacy of follow up of cohorts **(Outcome)** | | 0 | follow up 6955 but no description of those lost |

**Wu ZY, et al. (b) 2021**

| Study type | Cohort study | | |
| --- | --- | --- | --- |
| Participants | Adults undergoing annual health examinations.  Sample size: 8473  Mean age in years: 44.67±9.80  Gender: 4816males/3657females  Location: China | | |
| Outcomes | Main study outcome: investigate whether the triglyceride glucose index increases the risk of carotid atherosclerosis incidence.  Available outcomes: different level triglyceride glucose index and incidence of carotid atherosclerosis. | | |
| ***Risk of bias*** | | | |
| **Bias** | | **Authors’ judgment** | **Support for judgment** |
| Representativeness of the exposed cohort (Selection) | | 1 | truly representative of the average Beijing in the community |
| Selection of the non exposed cohort **(Selection)** | | 1 | drawn from the same community as the exposed cohort |
| Ascertainment of exposure **(Selection)** | | 1 | secure record (eg laboratory examination) |
| Demonstration that outcome of interest was not present at start of study **(Selection)** | | 1 | yes (excluded participants with carotid plaques) |
| Comparability of cohorts on the basis of the design or analysis **(Comparability)** | | 2 | study controls for population(no coronary heart disease, stroke or malignant tumor), age, and other factors |
| Assessment of outcome **(Outcome)** | | 1 | clearly defined |
| Was follow up long enough for outcomes to occur **(Outcome)** | | 1 | yes (7years) |
| Adequacy of follow up of cohorts **(Outcome)** | | 0 | follow up 8473 but no description of those lost |

**Laura S, et al. 2016**

| Study type | Cohort study | | |
| --- | --- | --- | --- |
| Participants | first-time attendee outpatients 18-90 years old  Sample size: 5014  Age: NA  Gender: 3068males/1946females  Location: Spain | | |
| Outcomes | Main study outcome: the association between triglyceride glucose index and cardiovascular diseases.  Available outcomes: different level triglyceride glucose index and incidence of cardiovascular diseases (cerebrovascular diseases). | | |
| ***Risk of bias*** | | | |
| **Bias** | | **Authors’ judgment** | **Support for judgment** |
| Representativeness of the exposed cohort (Selection) | | 0 | truly representative of the average Spain in the community |
| Selection of the non exposed cohort **(Selection)** | | 1 | drawn from the same community as the exposed cohort |
| Ascertainment of exposure **(Selection)** | | 1 | secure record (eg laboratory examination) |
| Demonstration that outcome of interest was not present at start of study **(Selection)** | | 1 | yes (excluded participants with cardiovascular diseases) |
| Comparability of cohorts on the basis of the design or analysis **(Comparability)** | | 1 | study controls for population(no type 1 diabetes or latent autoimmune diabetes), age and other factors |
| Assessment of outcome **(Outcome)** | | 1 | clearly defined |
| Was follow up long enough for outcomes to occur **(Outcome)** | | 1 | yes (12years) |
| Adequacy of follow up of cohorts **(Outcome)** | | 0 | 650 lost to follow up but no description of those lost |

**Wang L, et al. 2020**

| Study type | Cohort study | | |
| --- | --- | --- | --- |
| Participants | Patients with diabetes and acute coronary syndrome  Sample size: 2531  Mean age in years: 66.3±6.8  Gender: 1415 males/1116females  Location: China | | |
| Outcomes | Main study outcome: the prognostic value of the triglyceride glucose index in patients with diabetes and acute coronary syndrome.  Available outcomes: different level triglyceride glucose index and incidence of cardiovascular diseases (non-fatal stroke). | | |
| ***Risk of bias*** | | | |
| **Bias** | | **Authors’ judgment** | **Support for judgment** |
| Representativeness of the exposed cohort (Selection) | | 1 | truly representative of the average Tianjin’s patients with diabetes and acute coronary syndrome in the community |
| Selection of the non exposed cohort **(Selection)** | | 0 | no description of the derivation of the non exposed cohort |
| Ascertainment of exposure **(Selection)** | | 1 | secure record (eg laboratory examination) |
| Demonstration that outcome of interest was not present at start of study **(Selection)** | | 0 | no |
| Comparability of cohorts on the basis of the design or analysis **(Comparability)** | | 1 | study controls for population (diabetes and acute coronary syndrome) and other factors |
| Assessment of outcome **(Outcome)** | | 1 | clearly defined |
| Was follow up long enough for outcomes to occur **(Outcome)** | | 1 | yes (3years) |
| Adequacy of follow up of cohorts **(Outcome)** | | 0 | 2531(89.9%) patients completed the 3-year clinical follow-up but no description of those lost |

**Wang AX, et al. (4) 2021**

| Study type | Cohort study | | |
| --- | --- | --- | --- |
| Participants | Community residents 40 years and older  Sample size: 5381  Age: median [IQR]: 52.48 (45.65–61.58)  Gender: 3219males/2162females  Location: China | | |
| Outcomes | Main study outcome: the association of the triglyceride glucose index with intra-cranial and extra-cranial artery stenosis.  Available outcomes: the relationship between the triglyceride glucose index and intra-cranial and extra-cranial artery stenosis. | | |
| ***Risk of bias*** | | | |
| **Bias** | | **Authors’ judgment** | **Support for judgment** |
| Representativeness of the exposed cohort **(Selection)** | | 0 | somewhat representative of the average Chinese adults in the community |
| Selection of the non exposed cohort **(Selection)** | | 1 | drawn from the same community as the exposed cohort |
| Ascertainment of exposure **(Selection)** | | 1 | secure record (eg laboratory examination) |
| Demonstration that outcome of interest was not present at start of study **(Selection)** | | 1 | yes |
| Comparability of cohorts on the basis of the design or analysis **(Comparability)** | | 2 | study controls for population (free of coronary artery disease, transient ischemic attack, and stroke), age and other factors |
| Assessment of outcome **(Outcome)** | | 1 | clearly defined |
| Was follow up long enough for outcomes to occur **(Outcome)** | | 1 | yes (2 years) |
| Adequacy of follow up of cohorts **(Outcome)** | | 0 | 3447(intra-cranial artery stenosis) and 1853(extra-cranial artery stenosis) patients completed the 2-year follow-up but no description of those lost |

**Chen L, et al. 2022**

| Study type | Cohort study | | |
| --- | --- | --- | --- |
| Participants | Type 2 diabetes mellitus with underwent off-pump coronary artery bypass grafting  Sample size:1578  Mean age in years: 62.9 ± 8.0  Gender: 1116 males/161females  Location: China | | |
| Outcomes | Main study outcome: association between triglyceride-glucose index and 2-year adverse cardiovascular and cerebrovascular events.  Available outcomes: the relationship between the triglyceride glucose index and stroke. | | |
| ***Risk of bias*** | | | |
| **Bias** | | **Authors’ judgment** | **Support for judgment** |
| Representativeness of the exposed cohort **(Selection)** | | 1 | truly representative of the average Beijing’s patients with type 2 diabetes |
| Selection of the non exposed cohort **(Selection)** | | 1 | drawn from the same disease as the exposed cohort |
| Ascertainment of exposure **(Selection)** | | 1 | secure record (eg laboratory examination) |
| Demonstration that outcome of interest was not present at start of study **(Selection)** | | 1 | yes |
| Comparability of cohorts on the basis of the design or analysis **(Comparability)** | | 1 | study controls for population(type 2 diabetes), extreme body mass index and other factors |
| Assessment of outcome **(Outcome)** | | 1 | clearly defined |
| Was follow up long enough for outcomes to occur **(Outcome)** | | 1 | yes (2years) |
| Adequacy of follow up of cohorts **(Outcome)** | | 0 | no statement |

**Hu LL, et al. 2022**

| Study type | Cohort study | | |
| --- | --- | --- | --- |
| Participants | Elderly hypertensive patients  Sample size: 8487  Mean age in years: 68.77  Gender: 4008males/4479females  Location: China | | |
| Outcomes | Main study outcome: relationship between the triglyceride glucose index and the risk of first stroke.  Available outcomes: the relationship between the triglyceride glucose index and stroke. | | |
| ***Risk of bias*** | | | |
| **Bias** | | **Authors’ judgment** | **Support for judgment** |
| Representativeness of the exposed cohort **(Selection)** | | 1 | truly representative of the average jiangxi province patients with hypertensive patients |
| Selection of the non exposed cohort **(Selection)** | | 1 | drawn from the same community as the exposed cohort |
| Ascertainment of exposure **(Selection)** | | 1 | secure record (eg laboratory examination) |
| Demonstration that outcome of interest was not present at start of study **(Selection)** | | 1 | yes |
| Comparability of cohorts on the basis of the design or analysis **(Comparability)** | | 2 | study controls for population(free of stroke), age, medication history and other factors |
| Assessment of outcome **(Outcome)** | | 1 | clearly defined |
| Was follow up long enough for outcomes to occur **(Outcome)** | | 1 | yes (2years) |
| Adequacy of follow up of cohorts **(Outcome)** | | 0 | no statement |

**Guo QY, et al. 2022**

| Study type | Cohort study | | |
| --- | --- | --- | --- |
| Participants | Prediabetes and acute coronary syndrome patients.  Sample size: 2030  Mean age in years: 58.87 ± 10.27  Gender: 1505males/525females  Location: China | | |
| Outcomes | Main study outcome: influence of the triglyceride-glucose index on adverse cardiovascular and cerebrovascular events in prediabetic patients with acute coronary syndrome.  Available outcomes: the relationship between the triglyceride glucose index and stroke. | | |
| ***Risk of bias*** | | | |
| **Bias** | | **Authors’ judgment** | **Support for judgment** |
| Representativeness of the exposed cohort **(Selection)** | | 0 | somewhat representative of the prediabetes and acute coronary syndrome patients adults in the Beijing community |
| Selection of the non exposed cohort **(Selection)** | | 1 | drawn from the same hospital as the exposed cohort |
| Ascertainment of exposure **(Selection)** | | 1 | secure record (eg laboratory examination) |
| Demonstration that outcome of interest was not present at start of study **(Selection)** | | 0 | no (no statement) |
| Comparability of cohorts on the basis of the design or analysis **(Comparability)** | | 1 | study controls for population (prediabetes and acute coronary syndrome) and other factors |
| Assessment of outcome **(Outcome)** | | 1 | clearly defined |
| Was follow up long enough for outcomes to occur **(Outcome)** | | 1 | yes (1years) |
| Adequacy of follow up of cohorts **(Outcome)** | | 0 | no statement |

**Zhang Y, et al.(1) 2022**

| Study type | Cohort study | | |
| --- | --- | --- | --- |
| Participants | Nondiabetic patients with acute coronary syndrome with low-density lipoprotein cholesterol below 1.8 mmol/L  Sample size: 1655  Mean age in years: NA  Gender: 1223males/432females  Location: China | | |
| Outcomes | Main study outcome: high triglyceride-glucose index is associated with poor cardiovascular outcomes.  Available outcomes: the relationship between the triglyceride glucose index and Non-fatal stroke. | | |
| ***Risk of bias*** | | | |
| **Bias** | | **Authors’ judgment** | **Support for judgment** |
| Representativeness of the exposed cohort **(Selection)** | | 0 | somewhat representative of the Beijing's acute coronary syndrome adults |
| Selection of the non exposed cohort **(Selection)** | | 1 | drawn from the same hospital as the exposed cohort |
| Ascertainment of exposure **(Selection)** | | 1 | secure record (eg laboratory examination) |
| Demonstration that outcome of interest was not present at start of study **(Selection)** | | 0 | no (no excluded people with poor cardiovascular disease) |
| Comparability of cohorts on the basis of the design or analysis **(Comparability)** | | 1 | study controls for population (acute coronary syndrome undergoing coronary angiography) and other factors |
| Assessment of outcome **(Outcome)** | | 1 | clearly defined |
| Was follow up long enough for outcomes to occur **(Outcome)** | | 1 | yes (8 years) |
| Adequacy of follow up of cohorts **(Outcome)** | | 0 | no statement |

**Zhang Y, et al.(2) 2021**

| Study type | Cohort study | | |
| --- | --- | --- | --- |
| Participants | Patients with type 2 diabetes mellitus and acute myocardial infarction  Sample size: 1932  Mean age in years: 65.4±12.0  Gender: 1324males/608females  Location: China | | |
| Outcomes | Main study outcome: predictive effect of triglyceride-glucose index on clinical events.  Available outcomes: the relationship between the triglyceride glucose index and non-fatal stroke. | | |
| ***Risk of bias*** | | | |
| **Bias** | | **Authors’ judgment** | **Support for judgment** |
| Representativeness of the exposed cohort **(Selection)** | | 1 | somewhat representative of the Beijing's patients with type 2 diabetes mellitus and acute myocardial infarction |
| Selection of the non exposed cohort **(Selection)** | | 1 | drawn from the same hospital as the exposed cohort |
| Ascertainment of exposure **(Selection)** | | 1 | secure record (eg laboratory examination) |
| Demonstration that outcome of interest was not present at start of study **(Selection)** | | 1 | yes (without type 2 diabetes mellitus) |
| Comparability of cohorts on the basis of the design or analysis **(Comparability)** | | 1 | study controls for population (free of type 2 diabetes mellitus) and other factors |
| Assessment of outcome **(Outcome)** | | 1 | clearly defined |
| Was follow up long enough for outcomes to occur **(Outcome)** | | 1 | yes (7 years) |
| Adequacy of follow up of cohorts **(Outcome)** | | 0 | no statement |
