# Supplementary material for: Association between triglyceride glucose index and risk of cerebrovascular disease: systematic review and meta-analysis
Source: Cardiovasc Diabetol. 2022 Nov 2;21:226. doi: 10.1186/s12933-022-01664-9 (PMC9632026; doi:10.1186/s12933-022-01664-9)
Supplement: Supplementary file 5 — Supplementary Material 5: Table S3: The risk of bias for case-control/cross-sectional studies by the Newcastle-Ottawa scale (NOS) [file 12933_2022_1664_MOESM5_ESM.docx]

**Table S3** The risk of bias for case-control/cross-sectional studies by the Newcastle-Ottawa scale (NOS)

**Si S, et al. 2020**

| Study type | Case-control study | | |
| --- | --- | --- | --- |
| Participants | General adult population aged 40–69 years  Sample size: 273368  Age: NA  Gender: 156729males/116639females  Location: England | | |
| Outcomes | Main study outcome: different levels of triglyceride glucose index and the incidence of cerebrovascular diseases, triglyceride glucose index and relationships to demographics.  Available outcomes: different quantile triglyceride glucose index and incidence of cerebrovascular diseases. | | |
| ***Risk of bias*** | | | |
| **Bias** | | **Authors’ judgment** | **Support for judgment** |
| Is the case definition adequate(**Selection**) | | 1 | yes, with independent validation |
| Representativeness of the cases(**Selection**) | | 1 | consecutive or obviously representative series of cases |
| Selection of Controls(**Selection**) | | 1 | community controls |
| Definition of Controls(**Selection**) | | 1 | no history of disease (endpoint) |
| Comparability of cases and controls on the basis of the design or analysis(**Comparability**) | | 2 | study controls for age, gender and other factors |
| Ascertainment of exposure(**Exposure**) | | 0 | self report |
| Same method of ascertainment for cases and controls(**Exposure**) | | 1 | yes |
| Non-Response rate(**Exposure**) | | 0 | non respondents described |

**Nam Ki-W, et al. 2020**

| Study type | Cross-sectional study | | |
| --- | --- | --- | --- |
| Participants | Healthy population aged 40-79  Sample size: 2615  Mean age in years: 57  Gender: 1386males/1129females  Location: South Korea | | |
| Outcomes | Main study outcome: evaluated the relationship between the triglyceride glucose index and cerebral small vessel diseases.  Available outcomes: different quantile triglyceride glucose index and incidence of silent brain infarcts. | | |
| ***Risk of bias*** | | | |
| **Bias** | | **Authors’ judgment** | **Support for judgment** |
| Is the case definition adequate(**Selection**) | | 1 | yes, with independent validation |
| Representativeness of the cases(**Selection**) | | 1 | consecutive or obviously representative series of cases |
| Selection of Controls(**Selection**) | | 1 | community controls |
| Definition of Controls(**Selection**) | | 1 | no history of disease (endpoint) |
| Comparability of cases and controls on the basis of the design or analysis(**Comparability**) | | 2 | study controls for age, gender and other factors |
| Ascertainment of exposure(**Exposure**) | | 1 | secure record (laboratory examination) |
| Same method of ascertainment for cases and controls(**Exposure**) | | 1 | yes |
| Non-Response rate(**Exposure**) | | 0 | non respondents described |

**Wang AX, et al.(3) 2021**

| Study type | Cross-sectional study | | |
| --- | --- | --- | --- |
| Participants | Nondiabetic adults.  Sample size:4748  Median age (IQR): 51.84(45.32–60.40)  Gender: 2797males/1951females  Location: China | | |
| Outcomes | Main study outcome: association between the triglyceride-glucose index and carotid plaque stability.  Available outcomes: different quantile triglyceride glucose index and carotid plaque stability. | | |
| ***Risk of bias*** | | | |
| **Bias** | | **Authors’ judgment** | **Support for judgment** |
| Is the case definition adequate(**Selection**) | | 1 | yes, with independent validation |
| Representativeness of the cases(**Selection**) | | 1 | consecutive or obviously representative series of cases |
| Selection of Controls(**Selection**) | | 1 | community controls |
| Definition of Controls(**Selection**) | | 1 | no carotid plaque |
| Comparability of cases and controls on the basis of the design or analysis(**Comparability**) | | 1 | study controls for basic illness, gender and other factors |
| Ascertainment of exposure(**Exposure**) | | 1 | secure record (Laboratory examination) |
| Same method of ascertainment for cases and controls(**Exposure**) | | 1 | yes |
| Non-Response rate(**Exposure**) | | 0 | non respondents described |

**Shi WR, et al. 2020**

| Study type | Case-control study | | |
| --- | --- | --- | --- |
| Participants | Permanent residents over 40 years of age  Sample size: 10900  Age: NA  Gender: 4382males/6518females  Location: China | | |
| Outcomes | Main study outcome: the association between triglyceride glucose and prevalent ischemic stroke, the value of triglyceride glucose to optimize the risk stratification of ischemic stroke.  Available outcomes: different quantile triglyceride glucose index and carotid plaque stability. | | |
| ***Risk of bias*** | | | |
| **Bias** | | **Authors’ judgment** | **Support for judgment** |
| Is the case definition adequate(**Selection**) | | 1 | yes, with independent validation |
| Representativeness of the cases(**Selection**) | | 1 | consecutive or obviously representative series of cases |
| Selection of Controls(**Selection**) | | 1 | community controls |
| Definition of Controls(**Selection**) | | 1 | non-ischemic stroke |
| Comparability of cases and controls on the basis of the design or analysis(**Comparability**) | | 1 | study controls for age, gender and other factors |
| Ascertainment of exposure(**Exposure**) | | 1 | secure record (laboratory examination) |
| Same method of ascertainment for cases and controls(**Exposure**) | | 1 | yes |
| Non-Response rate(**Exposure**) | | 0 | non respondents described |

**Chiu H, et al. 2020**

| Study type | Cross-sectional study | | |
| --- | --- | --- | --- |
| Participants | Adults with type 2 diabetes  Sample size: 1990  Mean age in years: 64.0 ± 11.3  Gender: 856 males/1134females  Location: China | | |
| Outcomes | Main study outcome: the associations between the triglyceride glucose index and micro-angiopathies and macro-angiopathies.  Available outcomes: different quantile triglyceride glucose index and Micro- and Macro-angiopathies. | | |
| ***Risk of bias*** | | | |
| **Bias** | | **Authors’ judgment** | **Support for judgment** |
| Is the case definition adequate(**Selection**) | | 1 | yes, with independent validation |
| Representativeness of the cases(**Selection**) | | 0 | potential for selection biases (from the diabetes outpatient clinics of two hospitals) |
| Selection of Controls(**Selection**) | | 1 | hospital controls |
| Definition of Controls(**Selection**) | | 1 | non Micro- and Macro-angiopathies |
| Comparability of cases and controls on the basis of the design or analysis(**Comparability**) | | 0 | study controls for population. |
| Ascertainment of exposure(**Exposure**) | | 1 | secure record (laboratory examination) |
| Same method of ascertainment for cases and controls(**Exposure**) | | 1 | yes |
| Non-Response rate(**Exposure**) | | 0 | non respondents described |

**C. Irace, et al.(a) 2013**

| Study type | Cross-sectional study | | |
| --- | --- | --- | --- |
| Participants | Participants of the regional cardiovascular disease prevention campaign  Sample size: 330  Age: NA  Gender: 187males/143females  Location: Italy | | |
| Outcomes | Main study outcome: the association between carotid atherosclerosis and two simple markers of insulin resistance, i.e. homeostasis model assessment and triglyceride glucose Index.  Available outcomes: the associations between the triglyceride glucose index and carotid atherosclerosis. | | |
| ***Risk of bias*** | | | |
| **Bias** | | **Authors’ judgment** | **Support for judgment** |
| Is the case definition adequate(**Selection**) | | 1 | yes, with independent validation |
| Representativeness of the cases(**Selection**) | | 0 | potential for selection biases or not stated |
| Selection of Controls(**Selection**) | | 1 | community controls |
| Definition of Controls(**Selection**) | | 1 | no carotid atherosclerosis |
| Comparability of cases and controls on the basis of the design or analysis(**Comparability**) | | 0 | no description |
| Ascertainment of exposure(**Exposure**) | | 1 | secure record (laboratory examination) |
| Same method of ascertainment for cases and controls(**Exposure**) | | 1 | yes |
| Non-Response rate(**Exposure**) | | 0 | non respondents described |

**C. Irace, et al.(b) 2013**

| Study type | Cross-sectional study | | |
| --- | --- | --- | --- |
| Participants | Participants of the regional cardiovascular disease prevention campaign  Sample size:1432  Age: NA  Gender: 825males/607females  Location: Italy | | |
| Outcomes | Main study outcome: the association between carotid atherosclerosis and two simple markers of insulin resistance, i.e. homeostasis model assessment and triglyceride glucose Index (no plasma insulin).  Available outcomes: the associations between the triglyceride glucose index and carotid atherosclerosis. | | |
| ***Risk of bias*** | | | |
| **Bias** | | **Authors’ judgment** | **Support for judgment** |
| Is the case definition adequate(**Selection**) | | 1 | yes, with independent validation |
| Representativeness of the cases(**Selection**) | | 0 | potential for selection biases or not stated |
| Selection of Controls(**Selection**) | | 1 | community controls |
| Definition of Controls(**Selection**) | | 1 | no carotid atherosclerosis |
| Comparability of cases and controls on the basis of the design or analysis(**Comparability**) | | 0 | no description |
| Ascertainment of exposure(**Exposure**) | | 1 | secure record (laboratory examination) |
| Same method of ascertainment for cases and controls(**Exposure**) | | 1 | yes |
| Non-Response rate(**Exposure**) | | 0 | non respondents described |

**Alizargar J, et al. 2018**

| Study type | Case-control study | | |
| --- | --- | --- | --- |
| Participants | Community residents over 30 years old  Sample size: 276  Mean age in years: 56.15 ± 10.65  Gender: 156males/120females  Location: China | | |
| Outcomes | Main study outcome: evaluate the carotid plaque presence between hypertensive and normotensive individuals, the relationship between the triglyceride glucose index and carotid atherosclerosis.  Available outcomes: the relationship between the triglyceride glucose index and carotid plaque. | | |
| ***Risk of bias*** | | | |
| **Bias** | | **Authors’ judgment** | **Support for judgment** |
| Is the case definition adequate(**Selection**) | | 1 | yes, with independent validation |
| Representativeness of the cases(**Selection**) | | 0 | potential for selection biases or not stated |
| Selection of Controls(**Selection**) | | 1 | community controls |
| Definition of Controls(**Selection**) | | 1 | no carotid plaque |
| Comparability of cases and controls on the basis of the design or analysis(**Comparability**) | | 1 | study controls for age, population and other factors |
| Ascertainment of exposure(**Exposure**) | | 1 | secure record (laboratory examination) |
| Same method of ascertainment for cases and controls(**Exposure**) | | 1 | yes |
| Non-Response rate(**Exposure**) | | 0 | non respondents described |

**Zhao S, et al. 2019**

| Study type | Cross-sectional study | | |
| --- | --- | --- | --- |
| Participants | Community residents 65 years and older  Sample size:2830  Mean age in years: 71.5±6.2  Gender: 1259males/1571females  Location: China | | |
| Outcomes | Main study outcome: the association of macrovascular and micro vascular damage with the triglyceride glucose index (carotid plaque).  Available outcomes: the relationship between the triglyceride glucose index and carotid plaque. | | |
| ***Risk of bias*** | | | |
| **Bias** | | **Authors’ judgment** | **Support for judgment** |
| Is the case definition adequate(**Selection**) | | 1 | yes, with independent validation |
| Representativeness of the cases(**Selection**) | | 1 | consecutive or obviously representative series of cases |
| Selection of Controls(**Selection**) | | 1 | community controls |
| Definition of Controls(**Selection**) | | 1 | no carotid plaque |
| Comparability of cases and controls on the basis of the design or analysis(**Comparability**) | | 1 | study controls for age, gender and other factors |
| Ascertainment of exposure(**Exposure**) | | 1 | secure record (laboratory examination) |
| Same method of ascertainment for cases and controls(**Exposure**) | | 1 | yes |
| Non-Response rate(**Exposure**) | | 0 | non respondents described |

**Wang AX, et al. (4) 2021**

| Study type | Cross-sectional study | | |
| --- | --- | --- | --- |
| Participants | Community residents 40 years and older  Sample size: 5381  Median age (IQR): 52.48 (45.65–61.58)  Gender: 3219males/2162females  Location: China | | |
| Outcomes | Main study outcome: the association of the triglyceride glucose index with intra-cranial and extra-cranial artery stenosis.  Available outcomes: the relationship between the triglyceride glucose index and intra-cranial and extra-cranial artery stenosis. | | |
| ***Risk of bias*** | | | |
| **Bias** | | **Authors’ judgment** | **Support for judgment** |
| Is the case definition adequate(**Selection**) | | 1 | yes, with independent validation |
| Representativeness of the cases(**Selection**) | | 1 | consecutive or obviously representative series of cases |
| Selection of Controls(**Selection**) | | 1 | community controls |
| Definition of Controls(**Selection**) | | 1 | no endpoint diseases |
| Comparability of cases and controls on the basis of the design or analysis(**Comparability**) | | 2 | study controls for age, population and other factors |
| Ascertainment of exposure(**Exposure**) | | 1 | secure record (laboratory examination) |
| Same method of ascertainment for cases and controls(**Exposure**) | | 1 | yes |
| Non-Response rate(**Exposure**) | | 0 | non respondents described |

**Zhang NN, et al. 2021**

| Study type | Cross-sectional study | | |
| --- | --- | --- | --- |
| Participants | Community residents 40 years and older  Sample size: 1938  Age: NA  Gender: 925males/1013females  Location: China | | |
| Outcomes | Main study outcome: acumulative value of triglyceride glucose and high-sensitivity C-reactive protein to identify asymptomatic intracranial arterial stenosis, as well as its severity and numerical burden.  Available outcomes: the relationship between the triglyceride glucose index and asymptomatic intracranial arterial stenosis. | | |
| ***Risk of bias*** | | | |
| **Bias** | | **Authors’ judgment** | **Support for judgment** |
| Is the case definition adequate(**Selection**) | | 1 | yes, with independent validation |
| Representativeness of the cases(**Selection**) | | 1 | consecutive or obviously representative series of cases |
| Selection of Controls(**Selection**) | | 1 | community controls |
| Definition of Controls(**Selection**) | | 1 | no history of disease (endpoint) |
| Comparability of cases and controls on the basis of the design or analysis(**Comparability**) | | 2 | study controls for age, population and other factors |
| Ascertainment of exposure(**Exposure**) | | 1 | secure record (laboratory examination) |
| Same method of ascertainment for cases and controls(**Exposure**) | | 1 | yes |
| Non-Response rate(**Exposure**) | | 0 | non respondents described |
